# Causal analysis between altered levels of interleukins and obstructive sleep apnea

**DOI:** 10.3389/fimmu.2022.888644

**Published:** 2022-07-27

**Authors:** Minhan Yi, Wangcheng Zhao, Quanming Fei, Yun Tan, Kun Liu, Ziliang Chen, Yuan Zhang

**Affiliations:** ^1^ Department of Respiratory Medicine, Xiangya Hospital, Central South University, Changsha, China; ^2^ School of Life Sciences, Central South University, Changsha, China; ^3^ National Clinical Research Center for Geriatric Disorders, Xiangya Hospital, Central South University, Changsha, China; ^4^ Xiangya School of Medicine, Central South University, Changsha, China; ^5^ School of Computer Science and Engineering, Central South University, Changsha, China

**Keywords:** obstructive sleep apnea, Mendelian randomization, inflammation, interleukin, IL-6

## Abstract

**Background:**

Inflammation proteins including interleukins (ILs) have been reported to be related to obstructive sleep apnea (OSA). The aims of this study were to estimate the levels for several key interleukins in OSA and the causal effects between them.

**Method:**

Weighted mean difference (WMD) was used to compare the expression differences of interleukins between OSA and control, and the changed levels during OSA treatments in the meta-analysis section. A two-sample Mendelian randomization (MR) was used to estimate the causal directions and effect sizes between OSA risks and interleukins. The inverse-variance weighting (IVW) was used as the primary method followed by several other MR methods including MR Egger, Weighted median, and MR-Robust Adjusted Profile Score as sensitivity analysis.

**Results:**

Nine different interleukins—IL-1β, IL-2, IL-4, IL-6, IL-8, IL-12, IL-17, IL-18, and IL-23—were elevated in OSA compared with control to varying degrees, ranging from 0.82 to 100.14 pg/ml, and one interleukin, IL-10, was decreased by 0.77 pg/ml. Increased IL-1β, IL-6, and IL-8 rather than IL-10 can be reduced in OSA by effective treatments. Further, the MR analysis of the IVW method showed that there was no significant evidence to support the causal relationships between OSA and the nine interleukins—IL-1β, IL-2, IL-4, IL-5, IL-6, IL-8, IL-10, IL-17, and IL-18. Among them, the causal effect of OSA on IL-5 was almost significant [estimate: 0.267 (−0.030, 0.564), *p =* 0.078]. These results were consistent in the sensitivity analysis.

**Conclusions:**

Although IL-1β, IL-2, IL-4, IL-6, IL-8, IL-12, IL-17, IL-18, and IL-23 were increasing and IL-10 was reducing in OSA, no significant causal relationships were observed between them by MR analysis. Further research is needed to test the causality of OSA risk on elevated IL-5 level.

## Introduction

Obstructive sleep apnea (OSA) is a widespread sleep disorder that affected about 9% to 38% of the whole population (1). OSA is caused by repeated constriction or collapse of the upper airway during sleep, resulting in intermittent hypoxia (IH), sleep fragmentation, disarrangement to physiological homeostasis, and more (2). The underlying pathophysiological mechanisms of OSA are complex and multifactorial, and the activated systematic inflammation has participated in these processes (3). Therefore, research studies to understand the association and even the causality between OSA and inflammation are necessary for this field, which may benefit prevention and treatment for OSA and related health outcomes.

Interleukins (IL) are a type of cytokine that has been involved in a range of inflammatory pathways (4), and several interleukins have been studied in the development and pathogenesis of OSA (5). It is reported that activated hypoxia-inducible factor-1 alpha (HIF-1α) and nuclear factor κb (NF-κb) can upregulate the expression of IL-1β and IL-6 in OSA (6–8). Previous studies have also reported that the levels of IL-6 and IL-8 were higher in patients with OSA and associated with apnea-hypopnea index (AHI) as well (9–11). Moreover, IL-1β, IL-10, IL-18, and several other interleukins were also researched in OSA, but there were still lacked consistent opinions and a comprehensive evaluation for them in OSA (12–16). Therefore, a pooled analysis including more interleukins with a larger sample size is needed to assess the interactions between interleukins and OSA.

Further, it is difficult to assess the causality between OSA and inflammation from previous observational studies due to the potential confounding biases and reverse causation that existed (17). Mendelian randomization (MR), which uses genetic variants as instrumental variable (IV) to assure causality between given exposure and outcome (18), may be a good option to explore causality between OSA and interleukins. Because the genetic variants are naturally randomized to offspring during conception, MR can avoid the limitations commonly seen in observational studies (19). Through a similar method, we have confirmed the causality of OSA on the elevated inflammation protein of C-reactive protein (20). With the progress of data-sharing, genome-wide association studies (GWASs) identified multiple relevant variants about several interleukins and OSA, which bring available IV for a powerful MR (21, 22). In this study, we used SNPs independently associated with exposure (OSA/interleukins) as IVs and explore the causal effects on corresponding outcome (interleukins/OSA).

In general, we first explored the association between the interleukins and OSA by comparing the expression differences of 10 interleukins between OSA and control and the effect of treatments on four interleukins in OSA. Then, we applied a MR analysis to estimate the causal relationships between nine interleukins and OSA risk of OSA on interleukin levels and of interleukin concentrations on OSA risk.

## Materials and methods

### Meta-analysis of the association between interleukins and OSA

We carefully conducted this comprehensive analysis following the guidelines of the Preferred Reporting Items for Systematic Reviews and Meta-Analysis (PRISMA) in this paper (23).

#### Data sources and search strategy

We searched PubMed, Embase, Web of Science, and Cochrane Library by two authors independently with the terms “OSA OR OSAS OR OSAHS OR (sleep apnea)” and “IL OR interleukin”, up to 4 December 2021. The references of the enrolled studies were manually evaluated by two researchers independently and disagreements were resolved through discussion.

#### Inclusion criteria and exclusion criteria

On the basis of PICOS (participants, interventions, controls, outcomes, and studies), we defined the inclusion criteria as follows.

For the section of comparing protein expression between patients with OSA and controls, we listed the criteria as follows: P: OSA was diagnosed by clinical diagnostic criteria as by questionnaire or polysomnography (PSG) with an AHI > 5 events/h in adults, and AHI > 1 events/h in children without age, sex, BMI, and detection method restrictions. I: Detection methods including enzyme-linked immunosorbent assay (ELISA), multiplex assays, quantitative sandwich enzyme immunoassay kits, and other methods were used to detect the inflammation levels. C: Control group defined by questionnaire or PSG with AHI < 5 events/h in adults and AHI < 1 events/h in children. O: Studies evaluating the expression of interleukin levels reported sufficient data in format as mean ± standard deviation (SD). Moreover, all measurement units about interleukins are expressed as pg/ml. S: All studies with available data, like case-control research.

For the section of comparing the interleukins changed levels during OSA treatments, we listed the criteria as follows: P: same as described above. I: Interventions contained continuous positive airway pressure (CPAP) used for least one month, surgery like adenotonsillectomy and adenoidectomy, mandibular advancement device (MAD) and other oral appliances etc. O: The studies included both pre-treatment and post-treatment protein expression data. S: All studies with available data, like randomized clinical trials, prospective cohort study etc.

In addition, the exclusion criteria were as follows: (1) duplicated publications and (2) no original research (reviews, letters, editorials, and conference abstract).

#### Data extraction and quality Assessments

The data were extracted by two investigators. Basic information involved the first author’s name, year of publication, region, diagnosis of participants, severity of patients with OSA, age, body mass index (BMI), gender distribution and AHI of both groups, detection method, sample sources, and interleukin measurements (mean ± SD). For protein levels change after treatments, we also extracted treatments follow-up period. Finally, the Newcastle-Ottawa Scale (NOS) (24) assessed the quality of the studies included in this analysis.

#### Data analysis

All data were analyzed in the Review Manager 5.3 (The Nordic Cochrane Centre, The Cochrane Collaboration, London, UK). As for continuous outcomes, the weighted mean differences (WMDs) were used as measures of effect size in this study. We calculate an *I²* statistic to estimate heterogeneity. If *p* < 0.05 or *I^2^* > 50%, then the data were pooled by random effect model, otherwise by fixed effect model. A sensitivity analysis was also conducted by removing one article, in turn, to see its effect on the *p* value. Finally, a funnel diagram was conducted to evaluate publication bias.

In detail, first, we explored whether protein levels of interleukins were different between OSA and non-OSA. Then, similar analysis was conducted between subgroups of OSA with different severity and controls: mild OSA (AHI 5 to <15), moderate OSA (AHI 15 to <30), severe OSA (AHI ≥30), and mixed OSA (no clear definition of severity or difference degrees of severity) (25). To avoid the overlapping of controls, when available data for OSA were from different severity subgroups, whereas data for controls were from the same population, we only used the severe OSA subgroup data for analysis in total group analysis.

Second, as for possible interleukins, we analyzed whether interleukin levels changed over time after treatments in OSA (post-treatments VS pre-treatments), containing treatments of CPAP, surgery, and MAD.

### Mendelian randomization of the causality between interleukins and OSA

#### GWAS data sources

We used a published GWAS summary statistics from FinnGen Study that contained 217,955 individuals with 16,761 patients with OSA from European (21). In this GWAS, OSA was diagnosed on the basis of the International Statistical Classification of Diseases (ICD) codes (ICD-10: G47.3, ICD-9: 3472A), which were based on subjective symptoms, clinical examination, and sleep records (AHI ≥ 5 or respiratory event index ≥ 5).

Recently, Ahola-Olli et al. conducted a large-scale mapping of protein concentrations loci, and ELISA was used to measure 41 cytokines in 8,293 participants in Young Finns Study (YFS) and FINRISK surveys from Finnish cities (22). The GWAS summary statistics for several interleukins in our MR were collected from this research. In addition, to verify the results, we also included another GWAS summary statistics with a larger sample for IL-6, IL-8, and IL-18, which contained 21,758 participants of European descent from the UK Biobank (26).

#### Selection of instrument variable

Two-sample MR analysis is based on three assumptions: First, the genetic variants being used as an IV are associated with the exposure. Second, the used IV is not associated with the common confounders of exposures and outcomes. Third, there is no independent pathway between the genetic variants and the outcome other than through exposure (27). Independent SNPs were selected as IV if they had been associated with OSA (*p* < 5 × 10^−8^) (21) or interleukins (most with *p* < 5 × 10^−6^ except IL-10 with *p* < 5 × 10^−8^ for and IL-18 with *p* < 5 × 10^−7^ in the study from Ahola-Olli et al. (22), and all with *p* < 5 × 10^−8^ for IL-6, IL-8, and IL-18 in replication analysis (26)) in the GWAS source studies and in pair-wise linkage disequilibrium with the distance of 1,000 kb and r^2^ < 0.1 referring to the European population.

#### Mendelian randomization analysis

For MR analysis, we used the method of inverse-variance weighting (IVW) as the primary outcome that assumed genetic instruments for each risk factor satisfy the IV assumptions (28). Other MR methods including MR Egger (29), Weighted median (30), and MR-Robust Adjusted Profile Score (RAPS) (31) were used to correct for any potential violations of the assumptions in the sensitivity analysis. These three analyses are performed as they operate in different ways and rely on different assumptions for valid inferences to assess the reliability of MR analyses. In addition, heterogeneity was analyzed by Cochran’s Q-test of IVW and MR Egger, and pleiotropy was tested by the intercept of MR Egger analysis.

## Results

### Characteristics for all included publications

**Figure 1:**
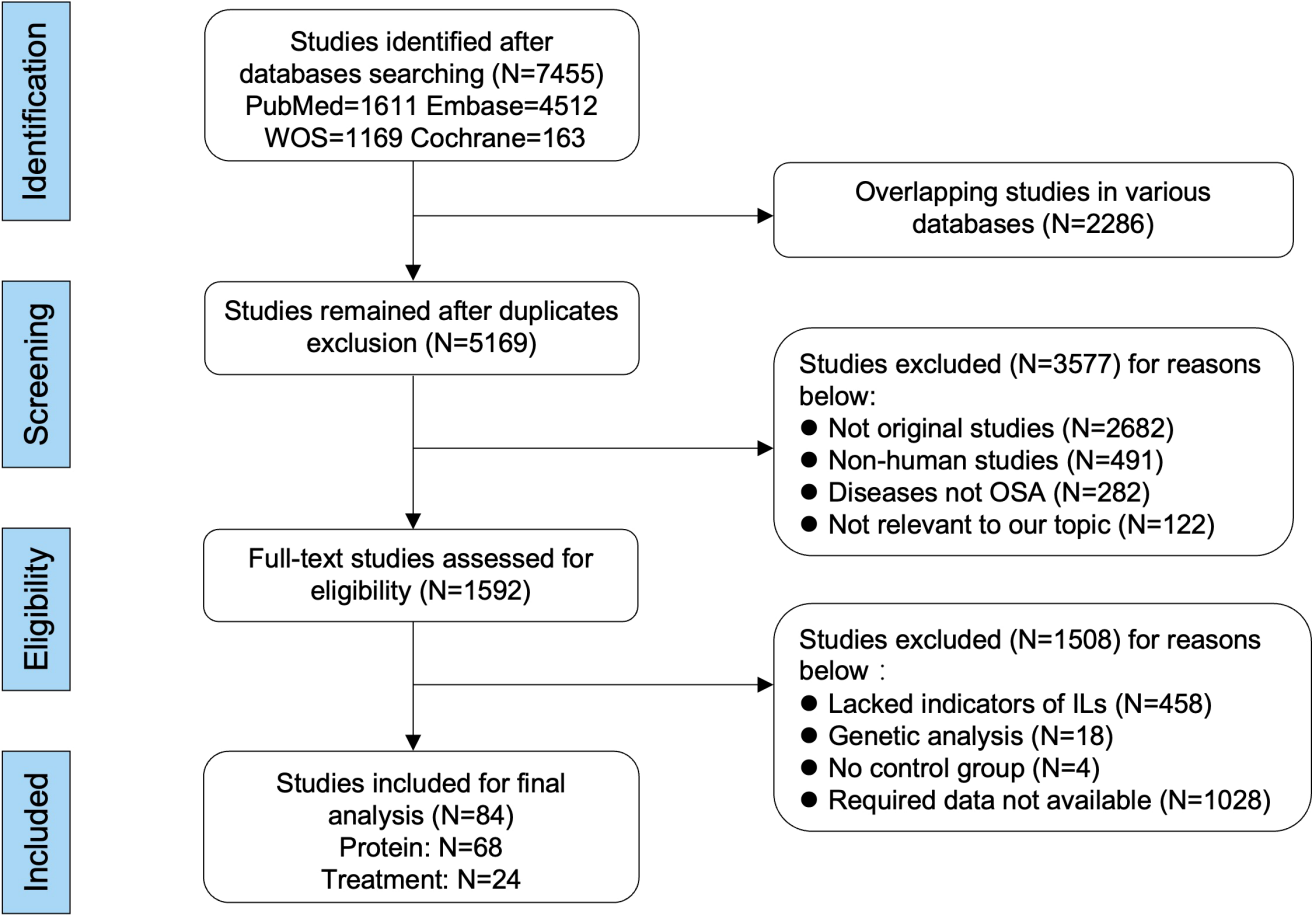
Flow diagram of literature selecting based on the inclusive and exclusive criteria. Among the included 84 publications, 68 studies were used for comparing the differences of interleukin levels between OSA and healthy control, and 24 studies were used for analyzing the changed levels of interleukins in patients with OSA responding to treatments. The format and unit of measurements were mean ± standard deviation and picograms per milliliter, respectively.

On the basis of the inclusion and exclusion criteria in the method section, a total of 84 articles were included for analysis (**Figure 1**). The characteristics of the 68 included articles about protein levels in patients with OSA and healthy control were shown in **Table 1** and **Supplemental Tables 1**, **2**. Moreover, the features of the 24 articles on responses of interleukin levels to treatments in OSA were shown in **Table 2** and **Supplemental Tables 3**, **4**. Each literature obtained a NOS score of at least 5.

**Table 1:** Characteristics of the included studies about interleukin levels in patients with OSA and control.

Study ID	Region	PMID	Researched protein	P/CNo.	NOS
Kotsiou O.S., 2022	Greece	34678476	IL-6	15/15	6
Bhatt S.P., 2021	India	34086720	IL-6, IL-8, IL-10, IL-17, IL-23	190/57	7
Bilal N., 2021	Turkey	32776303	IL-6, IL-8	30/30	8
Cheng J., 2021	China	34488706	IL-8	29/22	6
Chen B., 2021	China	33942365	IL-1β	67/30	8
Celikhisar H., 2020	Turkey	32454912	IL-1β	84/82	8
Chen V.G., 2020	Brazil	30213594	IL-1β, IL-4, IL-6, IL-8, IL-10	17/17	5
Dalesio N.M., 2020	US	31688081	IL-1β, IL-6, IL-10	7/18	5
Huang Y.S., 2020	China	32260590	IL-1β, IL-6, IL-10, IL-12, IL-17, IL-23	55/32	9
Ming H., 2019	China	30783447	IL-8	684/192	8
Tang T., 2019	China	30284175	IL-1β, IL-6, IL-18	120/127	7
Shi C., 2019	China	29453637	IL-2	15/15	8
Zhang D., 2019	China	30542936	IL-8	21/10	7
Bozic J., 2018	Croatia	29991422	IL-6	50/25	8
Kong Y., 2018	China	29843666	IL-1β, IL-6	50/40	5
Lu D., 2018	China	28707162	IL-6	35/22	6
Mônico-Neto M., 2018	Brazil	30093572	IL-6	447/211	5
Rogers V.E., 2018	US	29862666	IL-10, IL-12	20/7	5
Shalitin S., 2018	Israel	29305826	IL-6	9/29	6
Gamsiz-Isik H., 2017	Turkey	27858556	IL-1β	83/80	8
Jin F., 2017	China	28901415	IL-8	100/50	8
Said E.A., 2017	Oman	28830779	IL-2, IL-4, IL-8, IL-10, IL-12	22/21	6
Smith D.F., 2017	US	28204724	IL-6, IL-8	65/90	5
Su M., 2017	China	28500380	IL-4, IL-10	42/48	7
Toujani S., 2017	Tunisia	28469721	IL-17	92/30	8
Zhang Z., 2017	China	28367199	IL-2, IL-4, IL-6, IL-10	50/52	5
Archontogeorgis K., 2016	Greece	27843647	IL-8	64/20	7
Can M., 2016	Turkey	27665460	IL-23	12/27	6
Dogan D., 2016	Turkey	27346160	IL-6	39/12	8
Huang Y.S., 2016	China	27741107	IL-1β, IL-6, IL-10, IL-17, IL-23	47/32	6
Ifergane G., 2016	Israel	27073238	IL-6	21/22	8
Nizam N., 2016	Turkey	26232894	IL-6	39/13	7
Zychowski K.E., 2016	US	27693879	IL-6	8/7	6
Damiani M.F., 2015	Italy	26697221	IL-6	30/30	7
Leon-Cabrera S., 2015	Mexico	25944984	IL-10, IL-12	29/13	6
Nobili Y., 2015	Italy	26069285	IL-6	52/28	8
Sarinc Ulasli S., 2015	Turkey	25724552	IL-2, IL-10	28/20	6
Ye J., 2015	China	25860501	IL-4, IL-17	25/19	8
Zhang S.W., 2015	China	-	IL-18	66/25	8
Ciccone M.M., 2014	Italy	24481114	IL-6	80/40	7
Gileles-Hillel Alex, 2014	US	24991089	IL-6	75/129	9
Akinnusi M., 2013	US	23239459	IL-4, IL-6, IL-8, IL-10	25/18	8
Kurt O.K., 2013	Turkey	23783568	IL-6	48/37	7
Yang D., 2013	China	23567762	IL-6	25/25	7
Deboer M.D., 2012	US	21360253	IL-6	9/15	7
Medeiros C. A. M., 2012	Brazil	21916851	IL-1β, IL-6	50/15	7
Qian X., 2012	China	22527145	IL-6	30/40	6
Ye J., 2012	China	23345934	IL-6, IL-10, IL-17	44/20	9
Liu Z., 2011	China	-	IL-6	20/20	8
Kim J., 2010	Korea	20855682	IL-6, IL-8	37/22	6
Sahlman J., 2010	Finland	20040038	IL-1β, IL-6, IL-10	84/40	7
Steiropoulos P., 2010	Greece	20628509	IL-6	38/23	8
Ye L., 2010	China	20668869	IL-6	127/52	7
Li Y., 2009	China	18207457	IL-6, IL-10	68/22	7
Li C., 2009	China	19187612	IL-18	52/18	8
Antonopoulou S., 2008	Greece	18606530	IL-6	45/25	7
Arias M.A., 2008	Spain	18508832	IL-6	30/15	9
Constantinidis J., 2008	Greece	18317790	IL-1β, IL-6	13/12	7
Li Y., 2008	China	18311073	IL-6, IL-10	28/22	5
Nakra N., 2008	US	18762497	IL-6	24/9	7
Takahashi K.I., 2008	Japan	18199002	IL-6	41/12	7
Tomiyama H., 2008	Japan	18224268	IL-6, IL-1β	50/15	8
Chen J., 2007	China	-	IL-6	100/40	6
Tauman R., 2007	US	17171553	IL-6	78/33	8
Bao H.R., 2005	China	-	IL-8, IL-10	35/25	7
Ciftci T.U., 2004	Turkey	15381186	IL-6	43/22	6
Liu H., 2000	China	11215046	IL-6	22/16	6
Roytblat L., 2000	Israel	11225716	IL-6	11/9	5

IL, interleukin; P/C No., number of patients with OSA (P) and controls (C) in each group; NOS represents Newcastle-Ottawa Scale, which is used for article quality evaluation.

For the references for each study, please refer to **Supplemental Table 2**.

**Table 2:** Characteristics of included studies about interleukin changes during treatments.

Study ID	Region	PMID	No.	Researched protein	Period	NOS
Bilal N., 2021	Turkey	32776303	10	IL-6, IL-8	3M	6
Salman M.A., 2020	Egypt	–	79	IL-6	12M	6
Huang Y.S., 2020	China	32260590	55	IL-1β, IL-6, IL-10	6M	8
Chuang H.H., 2020	China	32093397	60	IL-1β, IL-8, IL-10	3M	8
Wang X., 2020	China	32799042	54	IL-6	3M	8
Borges Y.G., 2020	Brazil	31313021	18	IL-6, IL-10	2M	8
Recoquillon S., 2019	France	30366971	55	IL-6	2M	8
Campos-Rodriguez F., 2019	Spain	31314107	120	IL-6	3M	8
Tirado R., 2017	Spain	28283920	66	IL-1β, IL-6	12M	5
Jin F., 2017	China	28901415	100	IL-8	3M	9
Martínez-Cerón E., 2016	Spain	26910598	26	IL-6, IL-8	6M	8
Kheirandish-Gozal L., 2015	US	25801692	100	IL-6	< 6M	5
Arnardottir E.S., 2015	Iceland	25359691	177	IL-6	24M	8
Akinnusi M., 2013	US	23239459	25	IL-6, IL-8, IL-10	2M	6
Kezirian E.J., 2010	US	20564750	21	IL-6	3M	6
Eun Y.G., 2010	Korea	20632906	51	IL-6	1M	5
Ye L., 2010	China	20668869	10	IL-6	6M	7
Steiropoulos P., 2009	Greece	19413148	32	IL-6	6M	8
Li A.M., 2008	China	–	16	IL-6, IL-8	2–3M	8
Li Y., 2008 (a)*	China	18311073	33	IL-6, IL-10	2M	7
Li Y., 2008 (b)*			5			
Li Y., 2008 (c)*			2			
Takahashi K.I. 2008	Japan	18199002	27	IL-6	1M	8
Dorkova Z., 2008	Slovakia	18625666	16	IL-6	2M	8
Ryan S., 2006	Ireland	16840748	49	IL-6, IL-8, IL-10	1.5M	5
Bao H.R., 2005	China	-	10	IL-8, IL-10	6M	7

IL, interleukin; No., number of patients with OSA included in analysis. NOS represents Newcastle-Ottawa Scale, which is used for article quality evaluation. The period is measured in months. *: (a), (b), and (c) represent the different population from the same paper.

For the references for the included study, please refer to **Supplemental Table 4**.

### Interleukin levels and OSA severity

**Figure 2:**
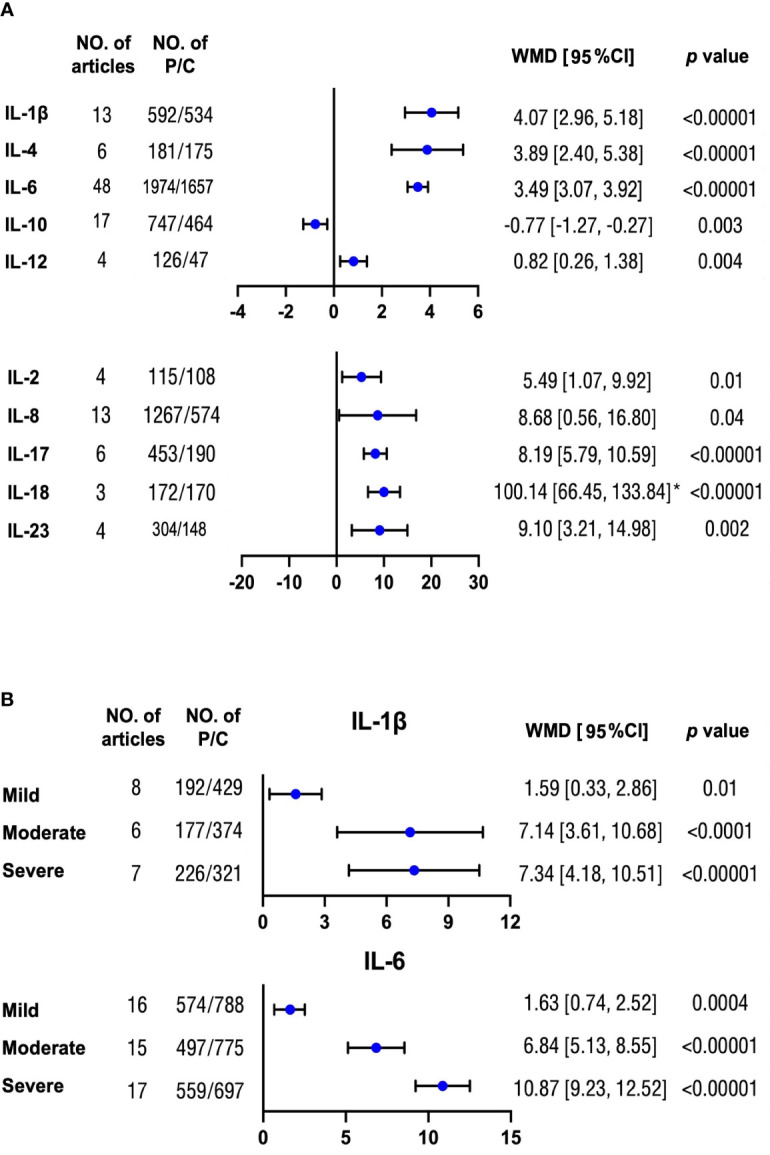
Mean differences of multiple interleukin levels between OSA and non-OSA subjects. **(A)** Concentration differences for several interleukin proteins were compared between patients with OSA and non-OSA controls. **(B)** Protein levels of IL-1β and IL-6 were analyzed between patients with OSA with mild, moderate, and severe severity and controls, respectively. The unit for all protein concentrations was presented as picograms per milliliter. Weighted mean difference (WMD) and 95% confidence interval (95% CI) were used for analysis. No. of articles, number of articles included for analysis in each group. No. of P/C, number of patients with OSA (P) and controls (C) were included for each group. *: The original protein value is 10 times higher than that shown in scale. The statistically different results with *p* < 0.05 were shown in blue point otherwise were in black point if *p* > 0.05. IL: interleukin.

In total, we compared the concentration differences of 10 interleukins between patients with OSA and controls. We found that nine different interleukins including IL-1β, IL-2, IL-4, IL-6, IL-8, IL-12, IL-17, IL-18, and IL-23 were elevated to varying degrees in patients with OSA compared with controls, with the differences ranging from 0.82 to 100.14 pg/ml. On the contrary, patients with OSA had lower IL-10 by 0.77 pg/ml than controls (**Figure 2A**, **Supplemental Figure 1**). The symmetric funnel plot illustrated no obvious publication bias (**Supplemental Figure 2**).

Moreover, there were increasing differences for IL-1β and IL-6 with serious OSA severity compared with controls (**Figure 2B** and **Supplemental Figure 3**). However, the trends for IL-8, IL-10, and IL-18 were not significant (**Supplemental Figure 4**). There was no obvious publication bias from the symmetric funnel plots. (**Supplemental Figure 5**)

### Interleukin levels and OSA treatment

**Figure 3:**
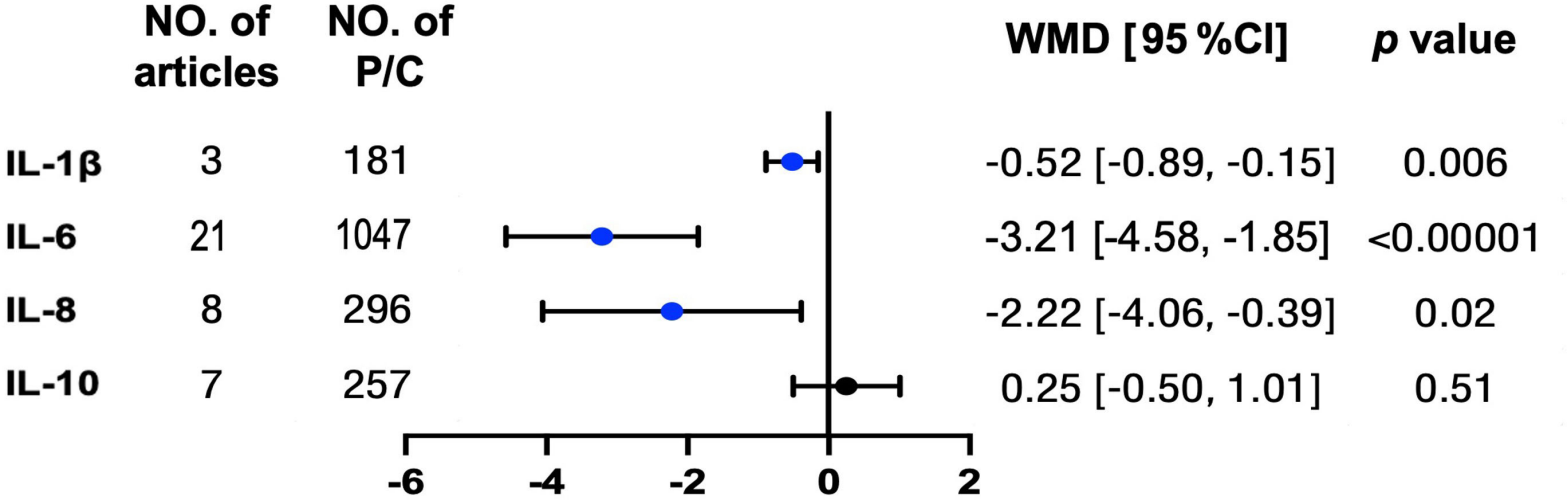
Typical inflammation but not anti-inflammation interleukins were reduced in patients with OSA after treatments. Concentrations for above interleukin proteins were tracked from pre-treatment to post treatment in patients with OSA. The unit for protein concentrations was presented as picograms per milliliter. Weighted mean difference (WMD) and 95% confidence interval (95% CI) were used for analysis. No. of articles, number of articles included for analysis in each group. No. of P, number of patients with OSA (P) included for each group. The statistically different results with *p* < 0.05 were shown in blue point otherwise were shown in black point if *p* > 0.05. IL, interleukin.

Afterward, we compared the level changes of several interleukins (IL-1β, IL-6, IL-8, and IL-10) from pre-treatment to post-effective OSA treatments in patients with OSA. Except for IL-10, the levels for the other three proteins all went down significantly, ranging from 0.52 to 3.21 pg/ml after treatments (**Figure 3** and **Supplemental Figure 6**). However, the level of IL-1β was sensitive to the study of Chuang et al. (32). The funnel plot illustrated the publication bias (**Supplemental Figure 7**).

### Causality analysis of OSA on interleukin levels

**Figure 4:**
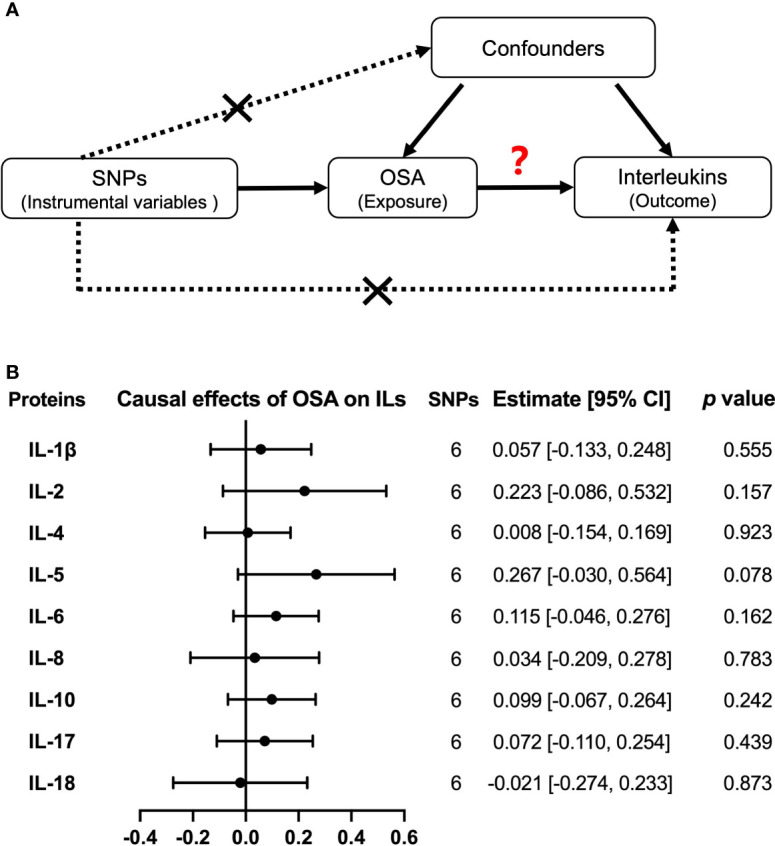
Causal relationships of OSA on interleukin levels by Mendelian randomization (MR) analysis. **(A)** The design of MR analysis was to explore the causal effects of OSA on interleukin levels: OSA as exposure, interleukins as outcomes, and SNPs independently associated with OSA from the used GWAS summary statistic were used as instrumental variables. In addition, other assumptions include the used SNPs is not associated with the confounders between exposure and outcome, and there is no independent pathway between the used SNPs and the outcome other than through exposure. **(B)** The presented results were performed by method of inverse-variance weighting (IVW). IL, interleukin.

With independent variants related to OSA as IV, we estimated the causal effects from OSA to nine interleukin levels by MR analysis (**Figure 4A**). As a result, we did not find any significant causal relations of OSA on IL-1β, IL-2, IL-4, IL-5, IL-6, IL-8, IL-10, IL-17, and IL-18 by the primary method of IVW but the result of IL-5 was almost significant [estimate: 0.267 (−0.030, 0.564), *p =* 0.078] (**Figure 4B**). Similar results were obtained by MR Egger, Weighted median, and MR RAPS (**Supplemental Table 5**). There was no evidence of heterogeneity in the IVW analysis. Moreover, the MR Egger showed that there was no directional pleiotropic effect across the all interleukin genetic variants (**Supplemental Table 5**).

Moreover, we used another large sample GWAS summary statistics of IL-6, IL-8, and IL-18 levels to verify the observed null associations of OSA on these hot researched interleukins. However, we still did not obtain any causal effects of OSA on IL-6 [estimate: 0.067, 95% CI = (−0.071, 0.205), *p =* 0.341], IL-8 [estimate: −0.069, 95% CI = (−0.200, 0.061), *p =* 0.297] and IL-18 [estimate: −0.043, 95% CI = (−0.163, 0.078), *p =* 0.488] by IVW (**Supplemental Table 6** and **Supplemental Figure 8A**)

### Causality analysis of interleukin levels on OSA risk

**Figure 5:**
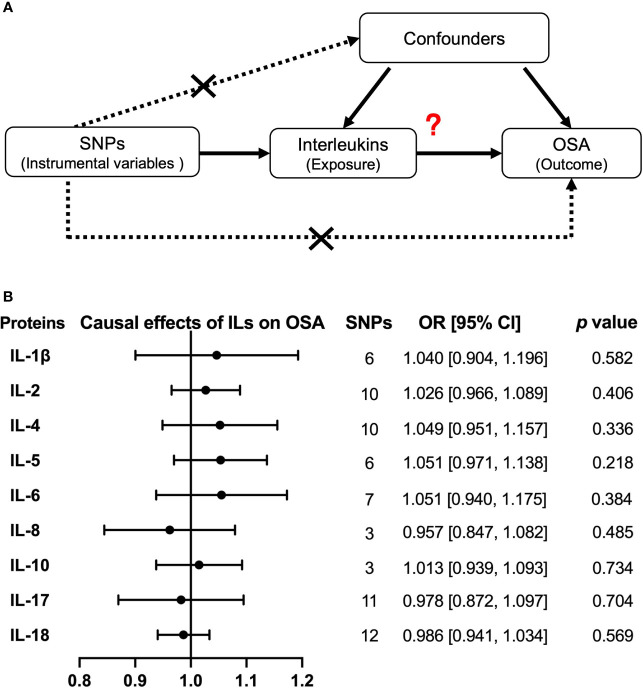
Causal relationships of interleukins on OSA risk by Mendelian randomization (MR) analysis. **(A)** The design of MR analysis was to explore the causal effects of interleukin levels on OSA risk: interleukins as exposure, OSA as outcomes, and SNPs independently associated with interleukins were used as instrumental variables. Other two assumptions are the same as described in the legend of **Figure 4A**. **(B)** The presented results were performed by method of inverse-variance weighting (IVW). IL, interleukin.

Then, we explored the causal effects of nine interleukins (IL-1β, IL-2, IL-4, IL-5, IL-6, IL-8, IL-10, IL-17, and IL-18) on OSA risk with each interleukin protein–related independent variants as IV (**Figure 5A**). The primary method of IVW model showed that there was no causal effect from interleukins to OSA risk (**Figure 5B**). Except for IL-17, there were no significant heterogeneity and horizontal pleiotropy in our results by IVW and MR Egger (**Supplemental Table 7**). Moreover, by another large sample GWAS, the causal effect of IL-6, IL-8, and IL-18 levels on OSA were still negative, with the OR of 0.853, 95% CI = (0.701, 1.039), and *p =* 0.114 for IL-6; OR of 0.982, 95% CI = (0.713, 1.353), and *p =* 0.912 for IL-8; and OR of 0.975, 95% CI = (0.941, 1.010), and *p =* 0.157 for IL-18 (**Supplemental Table 8** and **Supplemental Figure 8B**).

## Discussion

By combining meta-analysis and MR analysis, we discussed the relationship between a series of interleukin types and OSA. First, there are higher levels of IL-1β, IL-2, IL-4, IL-6, IL-8, IL-12, IL-17, IL-18, IL-23, and lower level of IL-10 in patients with OSA compared with controls. Especially, the expressions of IL-1β and IL-6 in OSA increased with OSA severity. Moreover, the levels of IL-1β and IL-6 along with IL-8 could be reduced by OSA treatments. However, no significant causal effects were observed either from OSA to nine different interleukin levels (IL-1β, IL-2, IL-4, IL-5, IL-6, IL-8, IL-10, IL-17, and IL-18) or from these interleukins to OSA risk.

The observed dysregulated interleukins provided clues for activated inflammations in OSA. Higher levels of monocytes derived IL-1β, IL-6, IL-8, and IL-12 supported monocytes activation in OSA, which was significantly increased in peripheral blood in response to chronic IH (33, 34). Further, we confirmed that levels of IL-1β and IL-6 were associated with OSA severity. In addition, IL-1β, IL-6, and IL-8 could be reduced by effective OSA interventions. These results suggested that dysfunction of monocyte and related inflammation pathways like NF-κb may be an important factor for the high inflammation in OSA (35). We also found that the alterations of several lymphocyte-derived interleukins such as IL-2, IL-4, and IL-12 in OSA indicated an imbalance in Th1/Th2 (13, 36). The levels for Th1 cytokines (IL-2 and IL-12) and Th2 cytokines (IL-4) were increased, whereas another Th2 cytokine IL-10 was decreased that may be due to reduced IL-10 content in γδT cells in OSA (37). Moreover, other interleukins including IL-17 and IL-23 have been recently emphasized. The increase of IL-17 and IL-23 implies a systematic inflammation in OSA (38). Under the regulation of IL-23, IL-17 can interact with other pro-inflammation interleukins like IL-1β and IL-6 and upregulate inflammation (39, 40). On the other side, mRNA levels of several inflammation interleukins (IL-1β, IL-6, IL-8, and IL-12) were upregulated in OSA which was consistent with our results (5, 41, 42). In addition, the dysregulation of interleukins may partly attribute to the gender differences, ethnicity, and others (43–45). All these changed interleukins in OSA emphasized the highly activated inflammation in OSA and indicated that several interleukin-related pathways are worthy of studying for OSA in the future.

Next, we used MR methods, which can overcome the influence of confounders, to explore causality between OSA and interleukins (46, 47). Interestingly, although we had no enough data to compare the concentration differences of IL-5 between patients with OSA and controls, we found that the causal effect of OSA on upregulated IL-5 was almost significant (*p =* 0.078, **Figure 5A**), which indicated the possibility that OSA may influence IL-5 levels in patients with OSA. IL-5 has been reported to be associated with several allergic diseases including asthma and allergic rhinitis, which were risk factors for developing OSA (48, 49). In addition, the release of OSA by adenotonsillectomy was associated with improvement in asthma symptoms (50). Thus, more attention is needed to pay for the association between the allergic reaction and OSA in future research. Further, we did not find causal effects of OSA on the above altered interleukin levels. In fact, several mechanisms aim to speculate about the causality from OSA to interleukins. IH in OSA can activate inflammatory pathways such as NF-κB, which enhanced the expression of several interleukins (34, 51). Another pathway of HIF-1α was also reported to participants in the activation of inflammasome in monocytes under IH (52). However, it has been reported by Arnardottir et al. that the association between OSA and IL-6 was found only in obese patients (53). Thus, a possibility for the observed inflammation status in OSA but no causality may also be explained by the results of OSA risk factors and related comorbidity, like obesity or hypertension and even the gender (54–56). In our reverse analysis about the causal effect of interleukins on OSA, we still got the null results by MR analysis. Some previous studies have inferred a mediator role of interleukins in OSA. For example, although related research is limited, a recent study further put forward that IL-6 is the mediation between obesity and OSA (46). According to the findings from bidirectional MR, more research studies from different models, such as associations of mRNA expression for each interleukin, are required to verify the causality between OSA and interleukins.

Despite the enlightening findings, this study did leave some limitations. First, there was relatively high heterogeneity in meta-analysis, which may be attributed to the variation of individual data. Second, the diagnosis of OSA GWAS was from medical electronic records, which had missed diagnosis. Because only people with clinical symptoms prefer to go hospital and those who have OSA but show no/mild symptoms may be mistakenly included in the control group. Third, the sample size of interleukin GWAS was relatively small, which may have a lower power to find enough related variants as IV. To complete this, we have replicated the analysis for IL-6, IL-8 and IL-18 with larger sample sizes. Thus, the associations of other interleukins and OSA are still needed to be studied.

In conclusion, there was a pervasively altered expression of interleukins including not only typical inflammation interleukins (IL-6 and IL-8) but also other interleukins (IL-1β, IL-2, IL-4, IL-10, IL-12, IL-17, IL-18, and IL-23). Among them, high IL-1β, IL-6, and IL-8 can be reduced by treatments in OSA. Finally, our MR results supported no causal relationships between OSA and above altered interleukins. Our results indicated that more research studies are required to answer the connective link between inflammation and OSA.

## Data availability statement

The original contributions presented in the study are included in the article/**Supplementary Material**. Further inquiries can be directed to the corresponding author.

## Author contributions

Conceptualization: MY and YZ. Data curation: WZ, MY, QF, YT, KL, ZC, and YZ. Data analysis: WZ, MY, QF, YT, KL, ZC, and YZ. Writing: WZ, MY, QF, YT, KL, ZC, and YZ. Funding acquisition: MY and YZ. Administration: MY and YZ. All authors contributed to the article and approved the submitted version.

## Funding

This research was supported by the National Natural Science Foundation of China (No. 82001357), the Hunan Provincial Natural Science Foundation of China (Nos. 2020JJ5951 and 2021JJ80079), the Youth Science Foundation of Xiangya Hospital (No. 2019Q17), the Degree and Postgraduate Education Reform Project of Central South University (No. 2021YJSKSA10), the Undergraduate Education Reform Project of Central South University (Nos. 2021CG065 and 2021CG068), and the Research Project of Laboratory Construction and Management of Central South University (No. 202120).

## Conflict of interest

The authors declare that the research was conducted in the absence of any commercial or financial relationships that could be construed as a potential conflict of interest.

## Publisher’s note

All claims expressed in this article are solely those of the authors and do not necessarily represent those of their affiliated organizations, or those of the publisher, the editors and the reviewers. Any product that may be evaluated in this article, or claim that may be made by its manufacturer, is not guaranteed or endorsed by the publisher.
