# Supplementary material for: Causal analysis between altered levels of interleukins and obstructive sleep apnea
Source: Front Immunol. 2022 Jul 27;13:888644. doi: 10.3389/fimmu.2022.888644 (PMC9363575; doi:10.3389/fimmu.2022.888644)
Supplement: Supplementary file 1 [file DataSheet_1.pdf]

Supplemental material 1: Supplemental Tables.

Supplemental Table 1. Characteristics of the included literature for Interleukins (ILs) protein level and OSA

Study ID	Groups	No.	ILs pg/ml mean±SD	AHI events/h	Age years	BMI kg/m2	Sex M/F	Sample source	Detection Methods
**IL-1β**									
Chen B. 2021	mild	20	7.29±3.43	11.09±3.04	43.80±9.61	28.45±5.90	14/6	serum	ELISA
	moderate	24	10.69±3.83	23.84±3.64	45.13±10.83	31.90±6.07	17/7		
	severe	23	13.17±5.50	74.80±21.24	44.78±11.64	33.23±6.16	18/5		
	control	30	2.67±1.89	3.06±1.35	44.77±10.67	26.15±3.55	21/9		
Celikhisar H. 2020	mild	28	11.80±7.04	/	/	/	28/0	serum	ELISA
	moderate	26	17.20±5.96	/	/	/	26/0		
	severe	30	23.74±2.98	/	/	/	30/0		
	control	82	1.70±0.21	/	/	/	82/0		
Chen V.G. 2020	mixed	17	102.03±112.98	/	/	/	/	Palatine tonsils	Milliplex-Human Cytokine/Chemokine reading kit
	control	17	97.26±139.67	/	/	/	/		
Dalesio N.M. 2020	severe	7	0.17±0.07	/	/	/	/	blood	/
	control	12	0.22±0.4	/	/	/	/		
Huang Y.S. 2020	mixed	55	1.51±2.25	15.71±22.60	7.67±2.64	16.83±4.03 (BMI score)	36/19	serum	ELISA
	control	32	0.42±1.38	0.46±0.28	7.02±0.65	17.44±3.08 (BMI score)	21/11		
Tang T. 2019	mixed	120	23.00±2.87	39.00±18.38	48.88±9.76	26.86±3.12	96/24	serum	ELISA
	mild	9	20.10±1.48	10.63±4.00	50.33±10.26	25.76±1.45	/		
	moderate	33	22.89±1.92	23.59±3.77	48.82±8.58	27.71±3.34	/		
	severe	78	24.92±2.86	48.78±14.81	48.73±10.51	27.66±3.14	/		
	control	127	20.86±2.56	3.31±1.09	47.37±9.12	22.46±3.29	100/27		

Kong Y. 2018	mixed	50	27.15±5.91	37.34±19.02	54.34±14.38	26.86±3.12	34/16	serum	ELISA
	mild	10	20.10±1.47	10.14±2.95	59.60±15.13	27.04±2.58	/		
	moderate	15	24.35±3.83	22.88±3.93	56.73±15.93	26.81±3.80	/		
	severe	25	29.15±5.91	49.11±14.96	52.27±13.54	27.28±3.16	/		
	control	40	21.17±1.70	3.31±1.09	50.42±8.35	22.26±3.54	31/9		
Gamsiz-Isik H. 2017	mixed	83	38.82±26.42	/	46.87±8.21	31.53±3.44	65/18	GCF	ELISA
	mild	16	34.35±24.2	/	/	/	/		
	moderate-severe	67	39.82±26.92	/	/	/	/		
	control	80	19.25±17.65	/	44.23±9.83	30.91±3.31	57/23		
Huang Y.S. 2016	mixed	47	0.36±0.16	9.13±1.67	7.84±0.56	16.95±0.47	30/17	serum	ELISA
	control	32	0.42±0.27	0.37±0.06	7.02±0.65	16.55±0.58	21/11		
Medeiros C. A. M. 2012	mild-moderate	15	0.00±0.0	/	62.62±9.0	24.50±3.8	11/4	serum	ELISA
	severe	35	1.22±4.6	/	65.0±7.2	25.9±4.1	20/15		
	control	15	0.00±0.0	/	62.50±8.4	25.81±4.04	6/9		
Sahlman J. 2010	mild	84	0.19±1.51	9.6±2.9	50.4±9.3	32.5±3.3	64/20	plasma	ELISA
	control	40	0.23±1.31	1.9±1.4	45.6±11.5	31.5±3.5	25/15		
Constantinidis J. 2008	mixed	13	0.28±0.11	3.4±0.3	/	33.4±1.5	13/0	serum	ELISA
	control	12	0.66±0.21	23.6±3.5	/	27.4±0.8	/		
Tomiyama H. 2008	mild	10	0.35±0.12	11±3	49±14	25.1±4.5	10/0	plasma	ELISA
	moderate	12	0.40±0.28	24±5	52±17	25.0±2.0	12/0		
	severe	28	0.43±0.33	62±22	52±11	28.3±4.3	28/0		
	control	15	0.33±0.16	/	53±10	24.3±2.5	15/0		
**IL-6**									
Kotsiou O. S. 2022	mixed	15	3.4±1.0	39.3±16.7	57.2 ± 8.2	40.8 ± 2.7	11/4	serum	two-site sandwich quantitative enzyme-linked immunosorbent assay using commercially available kits
	control	15	2.3±0.3	3.9±1.0	55.5 ± 13.7	38.7 ± 4.7	13/2		

Bhatt S. P. 2021	mixed	190	20.1±5.6	>=1	10.71±3.00	27.1±6.53	92/41	serum	/
	control	57	15.4±3.5	< 1	11.87±2.66	27.4±4.88	21/11		
Bilal N. 2021	mild	10	44.46±5.91	9.70±2.77	42.70±14.25	30.65±6.98	5/5	serum	ELISA
	moderate	10	42.74±2.49	20.24±4.40	47.00±9.63	30.66±7.76	8/2		
	severe	10	57.98±5.96	44.01±10.07	43.30±7.42	29.93±3.71	7/3		
	control	30	40.57±3.47	2.62±1.34	42.53±9.81	29.09±4.52	14/16		
Chen V.G. 2020	mixed	17	6.66±3.91	/	/	/	/	palatine tonsils	Milliplex-Human Cytokine/Chemokine reading kit
	control	17	7.22±4.96	/	/	/	/		
Dalesio N.M. 2020	severe	7	0.74±0.29	/	/	/	/	blood	/
	control	18	0.59±0.2	/	/	/	/		
Huang Y.S. 2020	mixed	55	1.59±1.58	15.71±22.60	7.67±2.64	16.83±4.03	36/19	serum	ELISA
	control	32	1.10±0.92	0.46±0.28	7.02±0.65	17.44±3.08	21/11		
Tang T. 2019	mixed	120	63.60±8.29	39.00±18.38	48.88±9.76	26.86±3.12	96/24	serum	ELISA
	mild	9	52.86±5.47	10.63±4.00	50.33±10.26	25.76±1.45	/		
	moderate	33	60.16±7.08	23.59±3.77	48.82±8.58	27.71±3.34	/		
	severe	78	66.29±7.62	48.78±14.81	48.73±10.51	27.66±3.14	/		
	control	127	59.16±12.27	3.31±1.09	47.37±9.12	22.46±3.29	100/27		
Kong Y. 2018	mixed	50	61.59±9.76	37.34±19.02	54.34±14.38	26.86±3.12	34/16	serum	ELISA
	mild	10	46.20±6.09	10.14±2.95	59.60±15.13	27.04±2.58	/		
	moderate	15	58.34±7.09	22.88±3.93	56.73±15.93	26.81±3.80	/		
	severe	25	64.76±9.05	49.11±14.96	52.27±13.54	27.28±3.16	/		
	control	40	54.46±9.43	3.31±1.09	50.42±8.35	22.26±3.54	31/9		
Bozic J. 2018	moderate	25	2.62±0.71	21.69±3.92	53.92±10.75	28.42±2.57	25/0	plasma	ELISA
	severe	25	3.58±1.55	48.26±18.10	52.04±13.11	29.30±2.74	25/0		
	control	25	1.26±0.69	/	52.52±10.18	27.78±2.23	25/0		
Lu D. 2018	mixed	35	58.49±8.03	/	48.63±1.80	28.21±0.51	/	serum	ELISA
	control	22	9.11±10.13	/	49.04±2.27	26.013±0.65	/		
	mild	201	10.5±0.9	10±0.4	49±12.3	27±0.2	91/110	blood	/

Mônico-Neto M. 2018	moderate	123	11±0.9	21±0.7	55±12.4	22±0.2	69/54		
	severe	123	10.1±0.8	45±1.3	58±13.4	28±0.2	70/53		
	control	211	9.1±0.9	3±0.3	42±10.4	26±0.2	61/150		
Shalitin S. 2018	mixed	9	4.56±2.92	/	/	/	/	plasma	ELISA
	control	29	2.83±1.54	/	/	/	/		
Smith D.F. 2017	mild	23	76.9±114.9	3.5±1.1	9.6±2.5	20.7±4.6	8/15	plasma	human multiplex cytokine assay
	moderate-severe	42	29.2±62.0	15.2±11.7	9.0±2.7	23.4±13.5	18/24		
	control	90	24.6±51.9	0.4±0.3	9.7±2.5	19.4±4.4	41/49		
Zhang Z. 2017	mixed	50	3.75±0.19	/	79.38 months	/	40/10	serum	flow cytometry
	control	52	2.54±0.17	/	77.13 months	/	33/19		
Huang Y.S. 2016	mixed	47	1.66±0.23	9.13±1.67	7.84±0.56	16.95±0.47	30/17	serum	ELISA
	control	32	1.10±0.18	0.37±0.06	7.02±0.65	16.55±0.58	21/11		
Dogan D. 2016	mixed	39	2.02±1.19	/	40.7±10.75	29.26±4.12	39/0	plasma	ELISA
	mild-moderate	12	1.21±0.41	/	36.9±6.5	29.46±4.73	12/0		
	severe	27	2.38±1.25	/	42.4±11.8	29.18±3.92	27/0		
	control	12	1.01±0.19	/	40.0±11.7	27.29±2.93	12/0		
Ifergane G. 2016	mixed	21	6.64±5.27	/	66.0±9.9	29.6±4.3	8/13	serum	ELISA
	control	22	3.14±2.05	/	66.1±13.1	26.8±4.3	5/17		
Nizam N. 2016	mild-moderate	17	18.1±13.1	17.24±7.90	49.88±11.47	31.85±5.32	9/8	saliva	ELISA
	severe	22	16.6±12.3	67.49±30.39	45.36±9.81	34.18±7.24	18/4		
	control	13	8.5±2.5	2.64±1.82	43.23±9.08	31.71±4.56	5/8		
	mild-moderate	17	99.1±42.5	17.24±7.90	49.88±11.47	31.85±5.32	9/8	serum	ELISA
	severe	22	99.2±41.1	67.49±30.39	45.36±9.81	34.18±7.24	18/4		
	control	13	82.4±27.2	2.64±1.82	43.23±9.08	31.71±4.56	5/8		

Zychowski K.E. 2016	severe	8	2.23±0.04	60.1±22.3	44.3±6.8	30.5±2.6	8/0	serum	electrochemiluminescence
	control	7	2.15±0.05	2.6±1.8	40.1±7.9	27.4±2.5	7/0		
Damiani M.F. 2015	moderate-severe	30	3.14±1.27	43.14±14.04	53.46±9.96	29.10±2.62	26/4	plasma	ELISA
	control	30	1.46±0.69	2.12±1.21	52.70±10.66	28.37±2.80	24/6		
Nobili Y. 2015	mixed	52	11.5±5.9	4.99±3.07	11.3±2.1	28.3±4.9	27/25	serum	ELISA
	mild	29	11.8±6.4	2.15±1.21	11.4±1.9	28.4±5.1	15/14		
	severe	23	10.5±4.3	8.77±3.05	11.2±2.5	28.5±5.0	12/11		
	control	28	11.0±5.6	0.58±0.30	11.7±1.9	26.4±5.9	18/10		
Ciccone M.M. 2014	mild	26	1.89±0.88	10.55±3.14	53.65±11.47	28.13±3.04	23/3	plasma	ELISA
	moderate-severe	54	3.33±1.35	45.13±16.08	52.33±10.19	28.8±3.03	45/9		
	control	40	1.5±0.67	2.11±1.14	52.27±10.52	28.24±2.7	34/6		
Gileles-Hillel Alex 2014	mixed	75	8±4.8	9±14.2	10.4±2.8	28±4.6	39/36	plasma	ELISA
	control	129	7.3±3.2	0.6±0.6	11±2.4	27.9±4.1	72/57		
Akinnusi M. 2013	moderate-severe	25	1.71±0.99	/	/	/	25/0	MNC#	ELISA
	control	18	1.36±0.92	1.9±1.6	52.3±9.3	29.5±5.1	18/0		
Kurt O.K. 2013	mixed	48	1.35±0.32	/	48.3±12.3	/	36/12	serum	ELISA
	control	37	1.31±0.30	/	43.1±14.1	/	22/15		
Yang D. 2013	mixed	25	1.89±1.29	24±17	54±7	27.39±2.91	23/2	plasma	ELISA
	control	25	1.0±0.12	3±1	53±7	26.27±1.9	23/2		
Medeiros C. A. M. 2012	mild-moderate	15	4.4±4.5	/	62.62±9.0	24.50±3.8	11/4	serum	ELISA
	severe	35	9.4±13.2	/	65.0±7.2	25.9±4.1	20/15		
	control	15	0.77±2.53	/	62.50±8.4	25.81±4.04	6/9		
Deboer M.D. 2012	mixed	9	1.69±1.10	13.5±11.9	14.2±1.66	/	4/5	serum	ELISA

	control	15	1.61±1.27	0.78±0.86	14.6±1.72	/	10/5		
Qian X. 2012	severe	30	89.56±36.19	/	45.0±9.0	29.4±2.1	30/0	serum	ELISA
	control	40	88.85±41.48	/	46.3±8.1	24.1±2.3	40/0		
Ye J. 2012	mixed	44	55.09±17.02	40.41±20.68	46.43±18.22	28.15±5.34	44/0	plasma	ELISA
	mild-moderate	23	56.20±22.41	23.12±6.93	44.90±20.64	28.55±6.29	23/0		
	severe	21	53.75±19.52	59.33±12.59	48.07±11.28	27.82±5.01	21/0		
	control	20	42.56±21.15	2.26±1.30	45.80±23.01	26.90±4.25	20/0		
Liu Z 2011	moderate-severe	20	1.62±0.43	48.8±10.2	41.2±5.2	28.3±2.8	11/9	serum	ELISA
	control	20	0.92±0.34	/	43.5±8.3	26.1±2.4	10/10		
Kim J. 2010	moderate	9	2.87±2.53	14.40±4.07	38±15.04	24.43±2.45	/	plasma	ELISA
	severe	28	3.57±2.74	52.71±22.23	42±10.77	28.69±4.05	/		
	control	22	3.02±2.44	1.25±1.25	26±6.91	23.88±2.30	/		
Sahlman J. 2010	mild	84	2.36±1.90	9.6±2.9	50.4±9.3	32.5±3.3	64/20	plasma	ELISA
	control	40	2.14±2.01	1.9±1.4	45.6±11.5	31.5±3.5	25/15		
Steiropoulos P. 2010	mixed	38	2.73±1.14	61±27	45.5±10.5	36.4±7.4	33/5	serum	ELISA
	control	23	2.36±1.41	5.3±3.2	43.7±6.7	34.5±3.7	17/6		
Ye L. 2010	mild	43	64.2±4.2	10.8±4.3	45±12	26.2±3.0	32/11	serum	ELISA
	moderate	39	121.2±9.9	29.5±5.2	47±10	26.6±4.8	31/8		
	severe	45	144.5±10.9	67.1±14.3	44±12	26.2±3.1	39/6		
	control	52	53.6±3.5	2.0±1.4	45±10	26.0±3.2	37/15		
Li Y. 2009	mild	22	8.4±1.2	14.1±3.5	48±12	25.7±4.2	15/7	EBC	ELISA
	moderate	22	13.9±1.9	29.7±5.5	44±13	28.8±5.3	18/4		
	severe	24	15.5±1.8	70.1±18.1	44±8	28.67±4.2	17/7		
	control	22	6.8±1.5	2.9±1.3	43±93	23.3±2.0	14/8		
	mild	22	55.2±9.6	14.1±3.5	48±12	25.7±4.2	15/7	serum	ELISA

	moderate	22	73.5±8.9	29.7±5.5	44±13	28.8±5.3	18/4		
	severe	24	83.4±6.4	70.1±18.1	44±8	28.67±4.2	17/7		
	control	22	37.5±10.9	2.9±1.3	43±93	23.3±2.0	14/8		
Constantinidis J. 2008	mixed	13	0.84±0.08	3.4±0.3	/	33.4±1.5	13/0	serum	ELISA
	control	12	0.42±0.08	23.6±3.5	/	27.4±0.8	/		
Antonopoulou S. 2008	mixed	45	0.53±0.3	39±25	52±12	33.5±7	37/8	EBC	ELISA
	control	25	0.21±0.2	<5	51±7	31±3	18/7		
Arias M.A. 2008	mixed	30	4.2±3.4	43.8±27.0	52±13	30.5±4.0	30/0	plasma	ELISA
	control	15	3.6±1.8	3.7±3.3	48±10	28.7±4.7	15/0		
Li Y. 2008	mixed	28	11.1±3.3	31.4±28.6	/	/	/	EBC	ELISA
	control	22	6.8±1.5	2.9±1.3	43±9	23.3±2.0	14/8		
	mixed	28	65.3±15.4	31.4±28.6	/	/	/	serum	ELISA
	control	22	37.5±10.9	2.9±1.3	43±9	23.3±2.0	14/8		
Nakra N. 2008	mixed	24	3.9±4.3	/	/	/	/		
	control	9	3.0±2.4	0.97 ± 0.44	12.3 ± 3.4	/	5/4		
Takahashi K.I. 2008	mixed	41	1.56±2.49	/	49.8±10.0	29.4±4.2	38/3	plasma	chemiluminescent enzyme immunoassay
	control	12	0.99±0.94	/	46.7±11.2	25.7±4.10	11/1		
Tomiyama H. 2008	mild	10	1.6±0.8	11±3	49±14	25.1±4.5	10/0	plasma	ELISA
	moderate	12	1.9±1.4	24±5	52±17	25.0±2.0	12/0		
	severe	28	2.4±1.6	62±22	52±11	28.3±4.3	28/0		
	control	15	1.5±0.6	/	53±10	24.3±2.5	15/0		
Chen J. 2007	mixed	100	143.12±33.52	/	/	/	67/33	serum	ELISA
	control	40	107.43±27.66	/	/	/	/		
Tauman R. 2007	mild	40	1.63±1.67	2.4±1.1	8.7±3.1	/	22/18	plasma	ELISA
	moderate-severe	38	2.26±2.7	17.3±11.4	8.2±3.0	/	21/17		

	control	33	1.12±0.70	0.19±0.25	7.5±1.9	/	17/16		
Ciftci T. U. 2004	mixed	43	11.71±9.8	33.19±24.99	49.6±9.1	31.86±4.11	43/0	serum	ELISA
	control	22	6.12±4.44	1.55±0.96	47.2±10.3	31.03±3.1	22/0		
Liu H. 2000	mixed	22	50.67±4.70	44.02±20.97	47.4±13.6	27.58±3.28	15/7	plasma	ELISA
	control	16	12.69±2.75	4.29±2.16	47.6±14.7	23.11±2.96	11/5		
	mixed	22	787.82±151.97	44.02±20.97	47.4±13.6	27.58±3.28	15/7	peripheral blood mononuclear cell (PBMC)	ELISA
	control	16	562.69±197.54	4.29±2.16	47.6±14.7	23.11±2.96	11/5		
Roytblat L. 2000	mixed	11	5.58±0.37	/	39.5±5	38.3±8	8/3	serum	ELISA
	control	9	7.69±5.06	/	35.5±9	36.6±8.2	0/9		
**IL-8**									
Bhatt S. P. 2021	mixed	190	8.29 ±5.2	>=1	10.71±3.00	27.1±6.53	92/41	serum	/
	control	57	4.98 ±3.67	< 1	11.87±2.66	27.4±4.88	21/11		
Bilal N. 2021	mild	10	106.83±11.56	9.70±2.77	42.70±14.25	30.65±6.98	5/5	serum	ELISA
	moderate	10	105.66±9.22	20.24±4.40	47.00±9.63	30.66±7.76	8/2		
	severe	10	126.31±11.73	44.01±10.07	43.30±7.42	29.93±3.71	7/3		
	control	30	100.12±5.13	2.62±1.34	42.53±9.81	29.09±4.52	14/16		
Cheng J. 2021	mixed	29	43.50 ± 7.22	/	42.3 ± 10.9	27.2 ± 2.8	18		
	control	22	45.65 ± 6.87	/	43.3 ± 9.5	24.3 ± 2.1	15		
Chen V.G. 2020	mixed	17	1088.77±1012.05	/	/	/	/	palatine tonsils	Milliplex-Human Cytokine/Chemokine reading kit
	control	17	664.44±565.13	/	/	/	/		
Ming H. 2019	mixed	684	34.6±7.1	31.15±9.12	/	/	446/238	serum	ELISA
	control	192	19.4±8.5	4.34±2.01	/	/	128/64		
Zhang D. 2019	mixed	21	229.71±175.61	/	/	/	21/0	sample of nasal lavage	ELISA
	control	10	69.40±26.70	/	/	/	10/0		
Jin F. 2017	mixed	100	40.72±1.60	38.011±8.040	55.284±7.128	26.746±3.500	82/18	plasma	ELISA

	control	50	13.28±3.15	3.623±1.537	56.131±6.210	25.196±2.449	37/13		
Said E.A. 2017	severe	22	75.2±32.9	/	40.4±8.6	/	18/4	plasma	CBA
	control	21	91.5±43.5	/	33.9±6.7	/	17/4		
Smith D.F. 2017	mild	23	24.9 ± 31.4	3.5±1.1	9.6±2.5	20.7±4.6	8/15	plasma	human multiplex cytokine assay
	moderate-severe	42	11.6 ± 16.7	15.2±11.7	9.0±2.7	23.4±13.5	18/24		
	control	90	9.8 ± 18.8	0.4±0.3	9.7±2.5	19.4±4.4	41/49		
Archontogeorgis K. 2016	mixed	64	31.79±20.09	/	51.78±11.55	36.34±13.18	52/12	serum	ELISA
	control	20	34.09±14.37	/	51.40±16.24	33.73±5.68	16/4		
Akinnusi M. 2013	moderate-severe	25	4.98±1.67	/	/	/	25/0	monocyte	ELISA
	control	18	4.57±1.453	1.9±1.6	52.3±9.3	29.5±5.1	18/0		
Kim J. 2010	moderate	9	11.90±2.65	14.40±4.07	38±15.04	24.43±2.45	/	plasma	ELISA
	severe	28	13.35±5.63	52.71±22.23	42±10.77	28.69±4.05	/		
	control	22	12.89±4.30	1.25±1.25	26±6.91	23.88±2.30	/		
Bao H.R. 2005	mixed	35	35.95±10.63	/	50±10	29.24±3.24	31/4	serum	ELISA
	control	25	22.27±7.62	/	50±12	28.04±2.27	22/3		
**IL-10**									
Bhatt S. P. 2021	mixed	190	2.62±0.39	>=1	10.71±3.00	27.1±6.53	92/41	serum	/
	control	57	2.10±0.28	< 1	11.87±2.66	27.4±4.88	21/11		
Chen V.G. 2020	mixed	17	13.08±11.77	/	/	/	/	palatine tonsils	Milliplex-Human Cytokine/Chemokine reading kit
	control	17	10.69±12.95	/	/	/	/		
Dalesio N.M. 2020	severe	7	0.59±0.17	/	/	/	/	blood	/
	control	18	0.46±0.16	/	/	/	/		
Huang Y.S. 2020	mixed	55	2.74±2.94	15.71±22.60	7.67±2.64	16.83±4.03	36/19	serum	ELISA
	control	32	2.10±1.41	0.46±0.28	7.02±0.65	17.44±3.08	21/11		

Rogers V.E. 2018	mixed	20	1.70±1.06	13.1±9.8	/	/	/	serum	ELISA
	control	7	2.03±1.76	0.8±0.3	/	/	/		
Said E.A. 2017	severe	22	0.5±0.3	/	40.4±8.6	/	18/4	plasma	CBA
	control	21	1.8±5.9	/	33.9±6.7	/	17/4		
Su M. 2017	mixed	42	261.71±18.55	8.68±4.56	/	/	/	plasma	ELISA
	control	48	338.24±25.20	2.40±2.75	/	/	/		
Zhang Z. 2017	mixed	50	3.45±0.19	/	79.38 months	/	40/10	serum	flow cytometry
	control	52	3.01±0.17	/	77.13 months	/	33/19		
Huang Y.S. 2016	mixed	47	2.62±0.39	9.13±1.67	7.84±0.56	16.95±0.47	30/17	serum	ELISA
	control	32	2.10±0.28	0.37±0.06	7.02±0.65	16.55±0.58	21/11		
Leon-Cabrera S. 2015	mixed	29	74.40±17.0	51.4±25.7	37 .2±11.4	45.2±8.4	4/25	serum	ELISA
	control	13	97.2±10.9	7.25±3.4	33.5±10.9	45.4±8.2	2/11		
Sarinc Ulasli S. 2015	mixed	28	34.7±12.4	21.2(5.5–73)	53.7±12.7	32.6±4.4	19/9	serum	ELISA
	control	20	32.5±14	2.2(0.5–4.2)	45.3±14	30.4±8	12/8		
Akinnusi M. 2013	moderate-severe	25	0.96±0.48	/	/	/	25/0	monocyte	ELISA
	control	18	0.89±0.43	1.9±1.6	52.3±9.3	29.5±5.1	18/0		
Ye J. 2012	mixed	44	47.84±10.72	40.41±20.68	46.43±18.22	28.15±5.34	44/0	plasma	ELISA
	mild-moderate	23	46.79±11.69	23.12±6.93	44.90±20.64	28.55±6.29	23/0		
	severe	21	49.06±8.51	59.33±12.59	48.07±11.28	27.82±5.01	21/0		
	control	20	52.17±23.18	2.26±1.30	45.80±23.01	26.90±4.25	20/0		
Sahlman J. 2010	mild	84	1.28±2.34	9.6±2.9	50.4±9.3	32.5±3.3	64/20	plasma	ELISA
	control	40	0.70±1.51	1.9±1.4	45.6±11.5	31.5±3.5	25/15		
Li Y. 2009	mild	22	46.7±4.6	14.1±3.5	48±12	25.7±4.2	15/7	serum	ELISA
	moderate	22	35.7±5.3	29.7±5.5	44±13	28.8±5.3	18/4		
	severe	24	24.6±5.1	70.1±18.1	44±8	28.67±4.2	17/7		
	control	22	50.1±6.9	2.9±1.3	43±93	23.3±2.0	14/8		

Li Y. 2008	mixed	28	38.3±13.6	31.4±28.6	/	/	/	EBC	ELISA
	control	22	56.8±6.8	2.9±1.3	43±9	23.3±2.0	14/8		
	mixed	28	37.5±12.4	31.4±28.6	/	/	/	serum	ELISA
	control	22	50.1±6.9	2.9±1.3	43±9	23.3±2.0	14/8		
Bao H.R. 2005	mixed	35	36.39±4.96	/	50±10	29.24±3.24	31/4	serum	ELISA
	control	25	42.06±4.09	/	50±12	28.04±2.27	22/3		
**IL-2**									
Shi C. 2019	mixed	15	59.98±8.57	33.30±21.68	44.46±12.56	27.00±2.21	12/3	sputum	ELISA
	control	15	36.58±6.20	2.07±0.83	41.33±9.44	24.65±1.86	11/4		
Said E.A. 2017	severe	22	0.3±0.2	/	40.4±8.6	/	18/4	plasma	CBA
	control	21	1.1±3.3	/	33.9±6.7	/	17/4		
Zhang Z. 2017	mixed	50	3.41±0.21	/	79.38 months	/	40/10	serum	flow cytometry
	control	52	3.38±0.19	/	77.13 months	/	33/19		
Sarinc Ulasli S. 2015	mixed	28	101.4±9.4	21.2 (5.5–73)	53.7±12.7	32.6±4.4	19/9	serum	ELISA
	control	20	97.2±10.2	2.2 (0.5–4.2)	45.3±14	30.4±8	12/8		
**IL-4**									
Chen V.G. 2020	mixed	17	6.67±2.37	/	/	/	/	palatine tonsils	Milliplex-Human Cytokine/Chemokine reading kit
	control	17	6.49±3.29	/	/	/	/		
Said E.A. 2017	severe	22	1.2±0.6	/	40.4±8.6	/	18/4	plasma	Cytometric beads array (CBA)
	control	21	5.6±20.4	/	33.9±6.7	/	17/4		
Su M. 2017	mixed	42	234.24±12.16	8.68±4.56	/	/	/	plasma	ELISA
	control	48	186.42±11.43	2.40±2.75	/	/	/		
Zhang Z. 2017	mixed	50	2.45±0.21	/	79.38 months	/	40/10	serum	flow cytometry
	control	52	1.21±0.22	/	77.13 months	/	33/19		
Ye J. 2015	mixed	25	36.72 ± 16.58	34.76 ± 15.28	6.45±2.84	1.28 ± 0.64, BMI z score	16/9	plasma	ELISA
	control	19	33.24 ± 15.69	0.38 ± 0.20	6.63±2.71,	1.25 ± 0.47, BMI z score	12/7		

Akinnusi M. 2013	moderate-severe	25	0.39±0.21	/	/	/	25/0	MNC#	ELISA
	control	18	0.36±0.20	1.9±1.6	52.3±9.3	29.5±5.1	18/0		
**IL-12**									
Huang Y.S. 2020	mixed	55	1.77±1.15	15.71±22.60	7.67±2.64	16.83±4.03	36/19	serum	ELISA
	control	6	0.97±0.75	/	/	/	/		
Said E.A. 2017	severe	22	1±2.6	/	40.4±8.6	/	18/4	plasma	CBA
	control	21	0±0.1	/	33.9±6.7	/	17/4		
Leon-Cabrera S. 2015	mixed	29	405.3±57.4	51.4±25.7	37 .2±11.4	45.2±8.4	4/25	serum	ELISA
	control	13	394±64.7	7.25±3.4	33.5±10.9	45.4±8.2	2/11		
Rogers V.E. 2018	mixed	20	4.88 ± 2.97	13.1±9.8	/	/	/	serum	ELISA
	control	7	5.37 ± 4.15	0.8±0.3	/	/	/		
**IL-17**									
Bhatt S. P. 2021	mixed	190	15.12±1.38	>=1	10.71±3.00	27.1±6.53	92/41	serum	/
	control	57	10.20±1.25	< 1	11.87±2.66	27.4±4.88	21/11		
Huang Y.S. 2020	mixed	55	13.78±7.18	15.71±22.60	7.67±2.64	16.83±4.03	36/19	serum	ELISA
	control	32	10.20±6.36	0.46±0.28	7.02±0.65	17.44±3.08	21/11		
Toujani, S. 2017	severe	92	20.3±3.9	55.7±17.8	52.3±12.7	36.2±6	48/44	serum	ELISA
	control	30	10.05±3	/	45.7±14.7	32±4.2	17/13		
Huang Y.S. 2016	mixed	47	15.12±1.38	9.13±1.67	7.84±0.56	16.95±0.47	30/17	serum	ELISA
	control	32	10.20±1.25	0.37±0.06	7.02±0.65	16.55±0.58	21/11		
Ye J. 2015	mixed	25	88.36±14.96	34.76±15.28	6.45 ± 2.84	1.28 ± 0.64, BMI z score	16/9	plasma	ELISA
	control	19	38.18±21.63	0.38±0.20	6.63 ± 2.71	1.25 ± 0.47, BMI z score	12/7		
Ye J. 2012	mixed	44	70.30±13.65	40.41±20.68	46.43±18.22	28.15±5.34	44/0	plasma	ELISA
	control	20	55.12±18.23	2.26±1.30	45.80±23.01	26.90±4.25	20/0		
**IL-18**									

Tang T. 2019	mixed	120	94.13±7.98	39.00±18.38	48.88±9.76	26.86±3.12	96/24	serum	ELISA
	mild	9	81.80±2.68	10.63±4.00	50.33±10.26	25.76±1.45	/		
	moderate	33	91.24±5.34	23.59±3.77	48.82±8.58	27.71±3.34	/		
	severe	78	96.77±7.63	48.78±14.81	48.73±10.51	27.66±3.14	/		
	control	127	89.72±12.71	3.31±1.09	47.37±9.12	22.46±3.29	100/27		
Zhang S.W. 2015	mild-moderate	32	15.57±5.09	/	42.31±5.31	24.84±1.58	24/8	serum	ELISA
	severe	34	19.26±9.87	/	44.74±9.02	25.04±2.52	25/9		
	control	25	10.58±2.02	/	/	/	/		
Li C. 2009	mild	16	352.08±76.32	11.10±3.89	50.03±15.03	28.37±4.20	16/0	plasma	ELISA
	moderate	18	600.17±83.91	29.78±5.19	47.11±12.76	27.85±2.52	18/0		
	severe	18	797.64±109.83	64.26±17.94	46.37±14.46	28.07±3.28	18/0		
	control	18	250.27±76.48	2.41±1.66	48.51±16.84	26.96±3.22	18/0		
**IL-23**									
Bhatt S. P. 2021	mixed	190	14.58±0.75	>=1	10.71±3.00	27.1±6.53	92/41	serum	/
	control	57	12.29±0.73	< 1	11.87±2.66	27.4±4.88	21/11		
Huang Y.S. 2020	mixed	55	20.14±7.08	15.71±22.60	7.67±2.64	16.83±4.03	36/19	serum	ELISA
	control	32	12.29±3.66	0.46±0.28	7.02±0.65	17.44±3.08	21/11		
Can M. 2016	severe	12	30.9±1.4	/	/	/	/	serum	ELISA
	control	27	6.9±1.3	1.6±1.2	49.6±11.7	28.8±4.6	21/6		
Huang Y.S. 2016	mixed	47	14.58±0.75	9.13±1.67	7.84±0.56	16.95±0.47	30/17	serum	ELISA
	control	32	12.29±0.73	0.37±0.06	7.02±0.65	16.55±0.58	21/11		

GCF: gingival crevicular fluid; ECLIA: electrochemiluminescence immunoassay; ELISA: enzyme-linked immunosorbent assay.

Supplemental Table 2. Detailed References Information in Interleukins (ILs) protein.

Study ID	References
Kotsiou O. S. 2022	1. Kotsiou OS, Siachpazidou DI, Pastaka C, et al. Association between Interleukin-6 and vitamin D serum levels in patients with obstructive sleep apnea syndrome and impact of long-term continuous positive airway pressure therapy on biomarker levels. *Respiratory Physiology and Neurobiology.* 2022;296.
Bhatt S. P. 2021	2. Bhatt SP, Guleria R, Kabra SK. Metabolic alterations and systemic inflammation in overweight/obese children with obstructive sleep apnea. *PLoS ONE.* 2021;16(6 June).
Bilal N. 2021	3. Bilal N, Kurutas EB, Orhan I, Bilal B, Doganer A. Evaluation of preoperative and postoperative serum interleukin-6, interleukin-8, tumor necrosis factor alpha and raftlin levels in patients with obstructive sleep apnea. *Sleep and Breathing.* 2021;25(2):819-826.
Cheng J. 2021	4. Cheng J, Xie Z, Wang S, et al. Cough hypersensitivity in patients with metabolic syndrome: a clinical finding and its possible mechanisms. *BMC Pulmonary Medicine.* 2021;21(1).
Chen B. 2021	5. Chen B, Liu YN, Ji L, et al. Elevated levels of interleukin-35 and interleukin-37 in adult patients with obstructive sleep apnea. *Journal of Clinical Laboratory Analysis.* 2021;35(6).
Celikhisar H. 2020	6. Celikhisar H, Ilkhan GD. Alterations in Serum Adropin, Adiponectin, and Proinflammatory Cytokine Levels in OSAS. *Canadian respiratory journal.* 2020;2020:2571283.
Chen V.G. 2020	7. Chen VG, Fonseca VMGD, Amaral JB, et al. Inflammatory markers in palatine tonsils of children with obstructive sleep apnea syndrome. *Brazilian Journal of Otorhinolaryngology.* 2020;86(1):23-29.
Dalesio N.M. 2020	8. Dalesio NM, Lee CKK, Hendrix CW, et al. Effects of Obstructive Sleep Apnea and Obesity on Morphine Pharmacokinetics in Children. *Anesthesia and analgesia.* 2020;131(3):876-884.
Huang Y.S. 2020	9. Huang YS, Chin WC, Guilleminault C, Chu KC, Lin CH, Li HY. Inflammatory factors: Nonobese pediatric obstructive sleep apnea and adenotonsillectomy. *Journal of Clinical Medicine.* 2020;9(4).
Ming H. 2019	10. Ming H, Tian A, Liu B, et al. Inflammatory cytokines tumor necrosis factor-, interleukin-8 and sleep monitoring in patients with obstructive sleep apnea syndrome. *Experimental and Therapeutic Medicine.* 2019;17(3):1766-1770.
Tang T. 2019	11. Tang T, Huang Q, Liu J, et al. Oxidative stress does not contribute to the release of proinflammatory cytokines through activating the Nod-like receptor protein 3 inflammasome in patients with obstructive sleep apnoea. *Sleep and Breathing.* 2019;23(2):535-542.
Shi C. 2019	12. Shi C, Liang S, Xu X, et al. Cough hypersensitivity in patients with obstructive sleep apnea hypopnea syndrome. *Sleep and Breathing.* 2019;23(1):33-39.
Zhang D. 2019	13. Zhang D, Xiao Y, Luo J, et al. Measurement of fractional exhaled nitric oxide and nasal nitric oxide in male patients with obstructive sleep apnea. *Sleep and Breathing.* 2019;23(3):785-793.
Bozic J. 2018	14. Bozic J, Borovac JA, Galic T, Kurir TT, Supe-Domic D, Dogas Z. Adropin and inflammation biomarker levels in male patients with obstructive sleep apnea: A link with glucose metabolism and sleep parameters. *Journal of Clinical Sleep Medicine.* 2018;14(7):1109-1118.
Kong Y. 2018	15. Kong Y, Li Z, Tang T, et al. The level of lipopolysaccharide-binding protein is elevated in adult patients with obstructive sleep apnea. *BMC Pulmonary Medicine.* 2018;18(1).
Lu D. 2018	16. Lu D, Abulimiti A, Wu T, Abudureyim A, Li N. Pulmonary surfactant-associated proteins and inflammatory factors in obstructive sleep apnea. *Sleep and Breathing.* 2018;22(1):99-107.

Mônico-Neto M. 2018	17. Mônico-Neto M, Moreira Antunes HK, Thomatieli Dos Santos RV, et al. Physical activity as a moderator for obstructive sleep apnoea and cardiometabolic risk in the EPISONO study. *European Respiratory Journal.* 2018;52(4).
Rogers V.E. 2018	18. Rogers VE, Bollinger ME, Tulapurkar ME, et al. Inflammation and asthma control in children with comorbid obstructive sleep apnea. *Pediatric Pulmonology.* 2018;53(9):1200-1207.
Shalitin S. 2018	19. Shalitin S, Deutsch V, Tauman R. Hepcidin, soluble transferrin receptor and IL-6 levels in obese children and adolescents with and without type 2 diabetes mellitus/impaired glucose tolerance and their association with obstructive sleep apnea. *Journal of Endocrinological Investigation.* 2018;41(8):969-975.
Gamsiz-Isik H. 2017	20. Gamsiz-Isik H, Kiyan E, Bingol Z, Baser U, Ademoglu E, Yalcin F. Does Obstructive Sleep Apnea Increase the Risk for Periodontal Disease? A Case-Control Study. *Journal of periodontology.* 2017;88(5):443-449.
Jin F. 2017	21. Jin F, Liu J, Zhang X, et al. Effect of continuous positive airway pressure therapy on inflammatory cytokines and atherosclerosis in patients with obstructive sleep apnea syndrome. *Molecular Medicine Reports.* 2017;16(5):6334-6339.
Said E.A. 2017	22. Said EA, Al-Abri MA, Al-Saidi I, et al. Altered blood cytokines, CD4 T cells, NK and neutrophils in patients with obstructive sleep apnea. *Immunology Letters.* 2017;190:272-278.
Smith D.F. 2017	23. Smith DF, Hossain MM, Hura A, et al. Inflammatory milieu and cardiovascular homeostasis in children with obstructive sleep apnea. *Sleep.* 2017;40(4).
Su M. 2017	24. Su MS, Xu L, Xu K, Zheng JS. Association of T lymphocyte immune imbalance and IL-10 gene polymorphism with the risk of obstructive sleep apnea in children with obesity. *Sleep and Breathing.* 2017;21(4):929-937.
Toujani S. 2017	25. Toujani S, Kaabachi W, Mjid M, Hamzaoui K, Cherif J, Beji M. Vitamin D deficiency and interleukin-17 relationship in severe obstructive sleep apnea-hypopnea syndrome. *Annals of Thoracic Medicine.* 2017;12(2):107-113.
Zhang Z. 2017	26. Zhang Z, Wang C. Immune status of children with obstructive sleep apnea/hypopnea syndrome. *Pakistan Journal of Medical Sciences.* 2017;33(1):195-199.
Archontogeorgis K.2016	27. Archontogeorgis K, Nena E, Tsigalou C, et al. Cystatin C Levels in Middle-Aged Patients with Obstructive Sleep Apnea Syndrome. *Pulmonary Medicine.* 2016;2016.
Can M. 2016	28. Can M, Uygur F, Tanriverdi H, Acikgoz B, Alper B, Guven B. Effect of continuous positive airway pressure (CPAP) therapy on IL-23 in patients with obstructive sleep apnea. *Immunologic research.* 2016;64(5-6):1179-1184.
Dogan D. 2016	29. Dogan D, Ocal N, Aydogan M, Tasci C, Tapan S, Yetkin S. Roles And Relationships Of Serum Ischemia-Modified Albumin In Obstructive Sleep Apnea In Comparison With Interleukin-6. *American Journal of Respiratory and Critical Care Medicine.* 2016;193.
Huang Y.S. 2016	30. Huang YS, Guilleminault C, Hwang FM, et al. Inflammatory cytokines in pediatric obstructive sleep apnea. *Medicine (United States).* 2016;95(41).
Ifergane G. 2016	31. Ifergane G, Ovanyan A, Toledano R, et al. Obstructive Sleep Apnea in Acute Stroke: A Role for Systemic Inflammation. *Stroke.* 2016;47(5):1207-1212.
Nizam N. 2016	32. Nizam N, Basoglu OK, Tasbakan MS, Lappin DF, Buduneli N. Is there an association between obstructive sleep apnea syndrome and periodontal inflammation? *Clinical oral investigations.* 2016;20(4):659-668.
Zychowski K.E. 2016	33. Zychowski KE, Sanchez B, Pedrosa RP, et al. Serum from obstructive sleep apnea patients induces inflammatory responses in coronary artery endothelial cells. *Atherosclerosis.* 2016;254:59-66.
Damiani M.F. 2015	34. Damiani MF, Zito A, Carratù P, et al. Obstructive Sleep Apnea, Hypertension, and Their Additive Effects on Atherosclerosis. *Biochem Res Int.* 2015;2015:984193.

Leon-Cabrera S. 2015	35. Leon-Cabrera S, Arana-Lechuga Y, Esqueda-León E, et al. Reduced Systemic levels of IL-10 are associated with the severity of obstructive sleep apnea and insulin resistance in morbidly obese humans. *Mediators of Inflammation.* 2015;2015.
Nobili Y. 2015	36. Nobili V, Alisi A, Cutrera R, et al. Altered gut-liver axis and hepatic adiponectin expression in OSAS:Novel mediators of liver injury in paediatric non-alcoholic fatty liver. *Thorax.* 2015;70(8):769-781.
Sarinc Ulasli S. 2015	37. Sarinc Ulasli S, Sarıaydın M, Gunay E, et al. Effects of nondipping pattern on systemic inflammation in obstructive sleep apnea. *Sleep and Breathing.* 2015;19(4):1185-1190.
Ye J. 2015	38. Ye J, Liu H, Li P, et al. CD4⁺ T-lymphocyte subsets in nonobese children with obstructive sleep apnea syndrome. *Pediatric Research.* 2015;78(2):165-173.
Zhang S.W. 2015	39. Zhang SW, Zhao GF, Liu YH, et al. MIF and IL - 18 serum levels in patients with OSAHS. *Journal of Dalian Medical University.* 2015;37(4):372-375.
Ciccone M.M. 2014	40. Ciccone MM, Scicchitano P, Zito A, et al. Correlation between inflammatory markers of atherosclerosis and carotid intima-media thickness in Obstructive Sleep Apnea. *Molecules (Basel, Switzerland).* 2014;19(2):1651-1662.
Gileles-Hillel Alex 2014	41. Gileles-Hillel A, Alonso-Álvarez ML, Kheirandish-Gozal L, et al. Inflammatory markers and obstructive sleep apnea in obese children: The NANOS study. *Mediators of Inflammation.* 2014;2014.
Akinnusi M. 2013	42. Akinnusi M, Jaoude P, Kufel T, El-Solh AA. Toll-like receptor activity in patients with obstructive sleep apnea. *Sleep and Breathing.* 2013;17(3):1009-1016.
Kurt O.K. 2013	43. Kurt OK, Tosun M, Talay F. Serum cardiotrophin-1 and IL-6 levels in patients with obstructive sleep apnea syndrome. *Inflammation.* 2013;36(6):1344-1347.
Yang D. 2013	44. Yang D, Liu Z, Luo Q. Plasma ghrelin and pro-inflammatory markers in patients with obstructive sleep apnea and stable coronary heart disease. *Medical Science Monitor.* 2013;19(1):251-256.
Deboer M.D. 2012	45. Deboer MD, Mendoza JP, Liu L, Ford G, Yu PL, Gaston BM. Increased systemic inflammation overnight correlates with insulin resistance among children evaluated for obstructive sleep apnea. *Sleep Breath.* 2012;16(2):349-354.
Medeiros C. A. M. 2012	46. Medeiros CAM, De Bruin VMS, Andrade GM, Coutinho WM, de Castro-Silva C, de Bruin PFC. Obstructive sleep apnea and biomarkers of inflammation in ischemic stroke. *Acta Neurologica Scandinavica.* 2012;126(1):17-22.
Qian X. 2012	47. Qian X, Yin T, Li T, et al. High levels of inflammation and insulin resistance in obstructive sleep apnea patients with hypertension. *Inflammation.* 2012;35(4):1507-1511.
Ye J. 2012	48. Ye J, Liu H, Zhang G, et al. The Treg/Th17 imbalance in patients with obstructive sleep apnoea syndrome. *Mediators of Inflammation.* 2012;2012.
Liu Z 2011	49. Liu Z, Xu Y, Hua Q, Wang Y, Liu R, Yang Z. Additive effects of obstructive sleep apnea syndrome and hypertension on inflammatory reaction. *African Journal of Biotechnology.* 2011;10(55):11738-11744.
Kim J. 2010	50. Kim J, Lee CH, Park CS, Kim BG, Kim SW, Cho JH. Plasma levels of MCP-1 and adiponectin in obstructive sleep apnea syndrome. *Archives of otolaryngology--head & neck surgery.* 2010;136(9):896 - 899.
Sahlman J. 2010	51. Sahlman J, Miettinen K, Peuhkurinen K, et al. The activation of the inflammatory cytokines in overweight patients with mild obstructive sleep apnoea. *J Sleep Res.* 2010;19(2):341-348.
Steiropoulos P. 2010	52. Steiropoulos P, Papanas N, Nena E, et al. Inflammatory markers in middle-aged obese subjects: Does obstructive sleep apnea syndrome play a role? *Mediators of Inflammation.* 2010;2010.

Ye L. 2010	53. Ye L, Ma GH, Chen L, et al. Quantification of circulating cell-free DNA in the serum of patients with obstructive sleep apnea-hypopnea syndrome. *Lung.* 2010;188(6):469-474.
Li Y. 2009	54. Li Y, Chongsuvivatwong V, Geater A, Liu A. Exhaled breath condensate cytokine level as a diagnostic tool for obstructive sleep apnea syndrome. *Sleep medicine.* 2009;10(1):95-103.
Li C. 2009	55. Li C, Zhang XL, Liu H, Wang ZG, Yin KS. Association among plasma interleukin-18 levels, carotid intima-media thickness and severity of obstructive sleep apnea. *Chinese Medical Journal.* 2009;122(1):24-29.
Antonopoulou S. 2008	56. Antonopoulou S, Loukides S, Papatheodorou G, Roussos C, Alchanatis M. Airway inflammation in obstructive sleep apnea: Is leptin the missing link? *Respiratory Medicine.* 2008;102(10):1399-1405.
Arias M.A. 2008	57. Arias MA, García-Río F, Alonso-Fernández A, et al. CPAP decreases plasma levels of soluble tumour necrosis factor-α receptor 1 in obstructive sleep apnoea. *European Respiratory Journal.* 2008;32(4):1009-1015.
Constantinidis J. 2008	58. Constantinidis J, Ereliadis S, Angouridakis N, Konstantinidis I, Vital V, Angouridaki C. Cytokine changes after surgical treatment of obstructive sleep apnoea syndrome. *European Archives of Oto-Rhino-Laryngology.* 2008;265(10):1275-1279.
Li Y. 2008	59. Li Y, Chongsuvivatwong V, Geater A, Liu A. Are biomarker levels a good follow-up tool for evaluating obstructive sleep apnea syndrome treatments? *Respiration.* 2008;76(3):317-323.
Nakra N. 2008	60. Nakra N, Bhargava S, Dzuira J, Caprio S, Bazzy-Asaad A. Sleep-disordered breathing in children with metabolic syndrome: The role of leptin and sympathetic nervous system activity and the effect of continuous positive airway pressure. *Pediatrics.* 2008;122(3):e634-e642.
Takahashi K.I. 2008	61. Takahashi KI, Chin K, Nakamura H, et al. Plasma thioredoxin, a novel oxidative stress marker, in patients with obstructive sleep apnea before and after nasal continuous positive airway pressure. *Antioxidants and Redox Signaling.* 2008;10(4):715-726.
Tomiyama H. 2008	62. Tomiyama H, Okazaki R, Inoue D, et al. Link between obstructive sleep apnea and increased bone resorption in men. *Osteoporosis International.* 2008;19(8):1185-1192.
Chen J. 2007	63. Chen J, Yang J, Yang WW, Wu H. Analysis of serum IL-6 and TNF-α levels in children with obstructive sleep apnea syndrome. *Chinese Journal of Evidence-Based Medicine.* 2007;7(7):547-549.
Tauman R. 2007	64. Tauman R, O'Brien LM, Gozal D. Hypoxemia and obesity modulate plasma C-reactive protein and interleukin-6 levels in sleep-disordered breathing. *Sleep and Breathing.* 2007;11(2):77-84.
Bao H.R. 2005	65. Bao HR, Yu Q, Liu XJ, Wang XY. Changes of serum interleukin-8 and monocyte chemoattractant protein-1 levels in patients with obstructive sleep apnea hypopnea syndrome. *Chinese Journal of Clinical Rehabilitation.* 2005;9(31):71-73.
Ciftci T. U. 2004	66. Ciftci TU, Kokturk O, Bukan N, Bilgihan A. The relationship between serum cytokine levels with obesity and obstructive sleep apnea syndrome. *Cytokine.* 2004;28(2):87-91.
Liu H. 2000	67. Liu H, Liu J, Xiong S, Shen G, Zhang Z, Xu Y. The change of interleukin-6 and tumor necrosis factor in patients with obstructive sleep apnea syndrome. *Journal of Tongji Medical University = Tong ji yi ke da xue xue bao.* 2000;20(3):200-202.
Roytblat L. 2000	68. Roytblat L, Rachinsky M, Fisher A, et al. Raised interleukin-6 levels in obese patients. *Obesity research.* 2000;8(9):673-675.

Supplemental Table 3. The expression of interleukins and AHI of included studies before and after treatment in OSA.

Study ID	ILs pg/ml mean±SD		AHI events/h	
	post	pre	post	pre
**IL-1β**				
Chuang H.H. 2020	0.3±0.2	0.8±0.8	2.4±3.1	11.5±13.6
Huang Y.S. 2020	0.35±0.29	1.51±2.25	2.98±4.35	15.71±22.60
Tirado R. 2017	0.46±0.47	0.67±0.61	9.14±9.71	33.8±26.1
**IL-6**				
Bilal N. 2021	55.17±3.74	57.98±5.96	21.50±4.94	44.01±10.07
Borges Y.G. 2020	7±4.2	8±3.1	/	/
Huang Y.S. 2020	1.22±1.21	1.59±1.58	2.98±4.35	15.71±22.60
Salman M.A. 2020	1.80±0.45	2.27±0.40	14.04±9.8	27.3±12.54
Wang X 2020	18.68±1.33	43.31±2.93	3.96±0.76	42.86±15.08
Campos-Rodriguez F. 2019	3.5 ± 2.6	3.6 ± 2.8	2.0 (0.7–4.1)	35.9 (24.2–50.1)
Recoquillon S. 2019	1.3±1.2	1.1 ±0.5	/	40.0 (34.0–51.0)
Tirado R. 2017	0.39±0.58	0.64±0.69	9.14±9.71	33.8±26.1
Martínez-Cerón E. 2016	8.0±4.6	11.1±4.7	/	35.6±23.4
Arnardottir E.S. 2015	2.8±2.0	2.4±1.7	/	50.8±20.3
Kheirandish-Gozal L. 2015	7.1±4.7	9.2±5.4	4.5±5.3	17.1±17.8
Akinnusi M. 2013	1.48±0.97	1.71±0.99	2.6±0.4	32.2±13.1
Kezirian E.J. 2010	2.00±1.30	2.96±2.74	/	/
Eun Y.G. 2010	2.4±3.4	7.6±10.4	/	31.1±21.3
Ye L. 2010	66.04±10.05	131.66±20.39	/	/
Steiropoulos P. 2009	2.46±1.08	2.71±1.27	/	61.12±28.29
Li A. M. 2008	0.2±0.3	0.3±0.4	1.4±2.0	28.7±27.7
Li Y. 2008 (a)	59.9±13.2	75.7±12.3	24.7±12.7	45.7±24.9
Li Y. 2008 (b)	51.6±23.8	71.4±15.4	12.9±15.6	38.6±19.3
Li Y. 2008 (c)	62.2±14.2	76.3±1.2	28.8±17.1	32.7±18.4
Dorkova Z. 2008	1.28±1.05	1.25±1.10	/	64.7±23.3
Takahashi K.I. 2008	0.634±0.619	1.68±2.87	/	48.2±14.8
Ryan S. 2006	4.46±16.39	5.13±22.65	7.35±5.35	41.60±45.06
**IL-8**				
Bilal N. 2021	112.87±8.99	126.31±11.73	21.50±4.94	44.01±10.07
Chuang H.H. 2020	4.1±2.2	6.4±3.6	2.4±3.1	11.5±13.6
Jin F. 2017	35.79±1.63	40.72±1.60	26.73±4.34	37.80±6.70
Martínez-Cerón E. 2016	9.7±4.0	10.7±3.3	/	35.6±23.4
Akinnusi M. 2013	4.73±1.56	4.98±1.67	2.6±0.4	32.2±13.1
Li A. M. 2008	1.1±0.6	1.9±1.0	1.4±2.0	28.7±27.7
Ryan S. 2006	4.68±1.56	5.48±1.53	7.35±5.35	41.60±45.06
Bao H.R. 2005	33.73±8.07	38.97±9.72	/	/
**IL-10**				
Borges Y.G. 2020	4±3.5	4 ±1.6	/	/
Chuang H.H. 2020	0.4±0.2	1.2±2.1	2.4±3.1	11.5±13.6
Huang Y.S. 2020	1.68±1.05	2.74±2.94	2.98±4.35	15.71±22.60

Akinnusi M. 2013	1.03±0.62	0.96±0.48	2.6±0.4	32.2±13.1
Li Y. 2008 (a)	44.9±7.3	33.8±8.7	24.7±12.7	45.7±24.9
Li Y. 2008 (b)	50.9±12.2	33.2±8.2	12.9±15.6	38.6±19.3
Li Y. 2008 (c)	44.3±5.0	36.1±13.2	28.8±17.1	32.7±18.4
Ryan S. 2006	1.08±0.60	1.31±0.49	7.35±5.35	41.60±45.06
Bao H.R. 2005	39.39±3.78	33.91±4.29	/	/

Supplemental Table 4. Detailed References Information in Interleukins (ILs) treantment.

Study ID	References
Bilal N. 2021	1. Bilal N, Kurutas EB, Orhan I, Bilal B, Doganer A. Evaluation of preoperative and postoperative serum interleukin-6, interleukin-8, tumor necrosis factor alpha and raftlin levels in patients with obstructive sleep apnea. *Sleep and Breathing.* 2021;25(2):819-826.
Salman M.A. 2020	2. Salman MA, Othman B, Salman AA, et al. Improvement of Obstructive Sleep Apnea-Hypopnea Syndrome in Morbidly Obese Patients Undergoing Laparoscopic Sleeve Gastrectomy: Prospective Study. *Bariatric Surgical Practice and Patient Care.* 2020;15(3):131-139.
Huang Y.S. 2020	3. Huang YS, Chin WC, Guilleminault C, Chu KC, Lin CH, Li HY. Inflammatory factors: Nonobese pediatric obstructive sleep apnea and adenotonsillectomy. *Journal of Clinical Medicine.* 2020;9(4).
Chuang H.H. 2020	4. Chuang HH, Huang CG, Chuang LP, et al. Relationships among and predictive values of obesity, inflammation markers, and disease severity in pediatric patients with obstructive sleep apnea before and after adenotonsillectomy. *Journal of Clinical Medicine.* 2020;9(2).
Wang X 2020	5. Wang X, Yue Z, Liu Z, et al. Continuous positive airway pressure effectively ameliorates arrhythmias in patients with obstructive sleep apnea-hypopnea via counteracting the inflammation. *American journal of otolaryngology - head and neck medicine and surgery.* 2020;41(6).
Borges Y.G. 2020	6. Borges YG, Ceia Cipriano LH, Aires R, et al. Oxidative stress and inflammatory profiles in obstructive sleep apnea: are short-term CPAP or aerobic exercise therapies effective? *Sleep and Breathing.* 2020;24(2):541-549.
Recoquillon S. 2019	7. Recoquillon S, Pépin JL, Vielle B, et al. Effect of mandibular advancement therapy on inflammatory and metabolic biomarkers in patients with severe obstructive sleep apnoea: a randomised controlled trial. *Thorax.* 2019;74(5):496 - 499.
Campos-Rodriguez F. 2019	8. Campos-Rodriguez F, Asensio-Cruz MI, Cordero-Guevara J, et al. Effect of continuous positive airway pressure on inflammatory, antioxidant, and depression biomarkers in women with obstructive sleep apnea: a randomized controlled trial. *Sleep.* 2019;42(10).
Tirado R. 2017	9. Tirado R, Masdeu MJ, Vigil L, et al. Impact of Bariatric Surgery on Heme Oxygenase-1, Inflammation, and Insulin Resistance in Morbid Obesity with Obstructive Sleep Apnea. *Obesity Surgery.* 2017;27(9):2338-2346.
Jin F. 2017	10. Jin F, Liu J, Zhang X, et al. Effect of continuous positive airway pressure therapy on inflammatory cytokines and atherosclerosis in patients with obstructive sleep apnea syndrome. *Molecular Medicine Reports.* 2017;16(5):6334-6339.
Martínez-Cerón E. 2016	11. Martínez-Cerón E, Barquiel B, Bezos AM, et al. Effect of Continuous Positive Airway Pressure on Glycemic Control in Patients with Obstructive Sleep Apnea and Type 2 Diabetes. A Randomized Clinical Trial. *American journal of respiratory and critical care medicine.* 2016;194(4):476 - 485.
Kheirandish-Gozal L. 2015	12. Kheirandish-Gozal L, Gileles-Hillel A, Alonso-Álvarez ML, et al. Effects of adenotonsillectomy on plasma inflammatory biomarkers in obese children with

	obstructive sleep apnea: A community-based study. *International Journal of Obesity.* 2015;39(7):1094-1100.
Arnardottir E.S. 2015	13. Arnardottir ES, Lim DC, Keenan BT, et al. Effects of obesity on the association between long-term sleep apnea treatment and changes in interleukin-6 levels: The Icelandic Sleep Apnea Cohort. *Journal of Sleep Research.* 2015;24(2):148-159.
Akinnusi M. 2013	14. Akinnusi M, Jaoude P, Kufel T, El-Solh AA. Toll-like receptor activity in patients with obstructive sleep apnea. *Sleep and Breathing.* 2013;17(3):1009-1016.
Kezirian E.J. 2010	15. Kezirian EJ, Malhotra A, Goldberg AN, White DP. Changes in obstructive sleep apnea severity, biomarkers, and quality of life after multilevel surgery. *Laryngoscope.* 2010;120(7):1481-1488.
Eun Y.G. 2010	16. Eun YG, Kim MG, Kwon KH, Shin SY, Cho JS, Kim SW. Short-term effect of multilevel surgery on adipokines and pro-inflammatory cytokines in patients with obstructive sleep apnea. *Acta Oto-Laryngologica.* 2010;130(12):1394-1398.
Ye L. 2010	17. Ye L, Ma GH, Chen L, et al. Quantification of circulating cell-free DNA in the serum of patients with obstructive sleep apnea-hypopnea syndrome. *Lung.* 2010;188(6):469-474.
Steiropoulos P. 2009	18. Steiropoulos P, Kotsianidis I, Nena E, et al. Long-term effect of continuous positive airway pressure therapy on infammation markers of patients with obstructive sleep apnea syndrome. *Sleep.* 2009;32(4):537-543.
Li A. M. 2008	19. Li AM, Lam HS, Chan MHM, et al. Inflammatory cytokines and childhood obstructive sleep apnoea. *Annals of the Academy of Medicine Singapore.* 2008;37(8):649-654.
Li Y. 2008	20. Li Y, Chongsuvivatwong V, Geater A, Liu A. Are biomarker levels a good follow-up tool for evaluating obstructive sleep apnea syndrome treatments? *Respiration.* 2008;76(3):317-323.
Takahashi K.I. 2008	21. Takahashi KI, Chin K, Nakamura H, et al. Plasma thioredoxin, a novel oxidative stress marker, in patients with obstructive sleep apnea before and after nasal continuous positive airway pressure. *Antioxidants and Redox Signaling.* 2008;10(4):715-726.
Dorkova Z. 2008	21. Dorkova Z, Petrasova D, Molcanyiova A, Popovnakova M, Tkacova R. Effects of continuous positive airway pressure on cardiovascular risk profile in patients with severe obstructive sleep apnea and metabolic syndrome. *Chest.* 2008;134(4):686-692.
Ryan S. 2006	23. Ryan S, Taylor CT, McNicholas WT. Predictors of elevated nuclear factor-kappaB-dependent genes in obstructive sleep apnea syndrome. *Am J Respir Crit Care Med.* 2006;174(7):824-830.
Bao H.R. 2005	24. Bao HR, Yu Q, Liu XJ, Wang XY. Changes of serum interleukin-8 and monocyte chemoattractant protein-1 levels in patients with obstructive sleep apnea hypopnea syndrome. *Chinese Journal of Clinical Rehabilitation.* 2005;9(31):71-73.

Supplemental Table 5. Causal effects of OSA on Interleukins (ILs) by Mendelian Randomization (MR) analysis.

Protein	Method	nsnp	estimate	se	p-value	Q	Q_df	Q_pvalue	intercept	intercept_se	intercept_pvalue
IL-1β	IVW	6	0.057362	0.097236	0.5552422	3.456783	5	0.6299368	NA	NA	NA
	MR Egger	6	-0.12399	0.395746	0.7697122	3.233305	4	0.5195685	0.017119	0.036212	0.661043
	Weighted median	6	0.025213	0.124132	0.8390492	NA	NA	NA	NA	NA	NA
	MR RAPS	6	0.058225	0.100417	0.562025	NA	NA	NA	NA	NA	NA
IL-2	IVW	6	0.223022	0.157516	0.1568123	7.913903	5	0.1610449	NA	NA	NA
	MR Egger	6	-0.10004	0.693946	0.8923423	7.482204	4	0.1124966	0.030505	0.0635	0.656033
	Weighted median	6	0.250252	0.173386	0.1489305	NA	NA	NA	NA	NA	NA
	MR RAPS	6	0.230525	0.128855	0.0736115	NA	NA	NA	NA	NA	NA
IL-4	IVW	6	0.007965	0.082416	0.9230095	5.003613	5	0.4154394	NA	NA	NA
	MR Egger	6	0.315915	0.341492	0.4072715	4.113981	4	0.3907999	-0.02907	0.031254	0.404994
	Weighted median	6	0.018458	0.107251	0.8633549	NA	NA	NA	NA	NA	NA
	MR RAPS	6	0.008137	0.084726	0.9234934	NA	NA	NA	NA	NA	NA
IL-5	IVW	6	0.267185	0.151621	0.0780374	7.126716	5	0.2113856	NA	NA	NA
	MR Egger	6	0.855424	0.609602	0.233203	5.709781	4	0.2218957	-0.05571	0.055919	0.375487
	Weighted median	6	0.345087	0.167521	0.0394026	NA	NA	NA	NA	NA	NA
	MR RAPS	6	0.275156	0.131279	0.0360856	NA	NA	NA	NA	NA	NA
IL-6	IVW	6	0.114943	0.082175	0.1618869	2.946751	5	0.7081952	NA	NA	NA
	MR Egger	6	0.217542	0.334886	0.5513675	2.846874	4	0.5837688	-0.00968	0.030631	0.767781
	Weighted median	6	0.14537	0.105171	0.1669029	NA	NA	NA	NA	NA	NA
	MR RAPS	6	0.116394	0.085241	0.1721066	NA	NA	NA	NA	NA	NA
IL-8	IVW	6	0.034224	0.124155	0.7828117	4.115647	5	0.5328888	NA	NA	NA
	MR Egger	6	0.018938	0.50862	0.9720828	4.114657	4	0.3907111	0.001445	0.046578	0.976739
	Weighted median	6	0.144775	0.159166	0.363042	NA	NA	NA	NA	NA	NA
	MR RAPS	6	0.034826	0.127981	0.7855289	NA	NA	NA	NA	NA	NA
IL-10	IVW	6	0.098857	0.084414	0.2415579	2.475217	5	0.7802227	NA	NA	NA
	MR Egger	6	0.33397	0.344049	0.386663	1.978308	4	0.7397488	-0.02217	0.031456	0.519743
	Weighted median	6	0.099318	0.104476	0.341792	NA	NA	NA	NA	NA	NA
	MR RAPS	6	0.099886	0.087545	0.2538836	NA	NA	NA	NA	NA	NA
IL-17	IVW	6	0.071913	0.092955	0.43915	6.087695	5	0.2977791	NA	NA	NA
	MR Egger	6	0.481763	0.368661	0.2613488	4.581895	4	0.332947	-0.03865	0.03371	0.315493
	Weighted median	6	0.123443	0.111973	0.270272	NA	NA	NA	NA	NA	NA
	MR RAPS	6	0.073814	0.086563	0.3938153	NA	NA	NA	NA	NA	NA
IL-18	IVW	6	-0.02058	0.129129	0.8733503	5.575699	5	0.3497166	NA	NA	NA
	MR Egger	6	-0.43719	0.540325	0.4638207	4.813337	4	0.306992	0.039408	0.049511	0.470625
	Weighted median	6	-0.07542	0.162104	0.6417381	NA	NA	NA	NA	NA	NA
	MR RAPS	6	-0.02109	0.125606	0.8666725	NA	NA	NA	NA	NA	NA

Causality of OSA on interleukins : causality was analyzed with OSA as exposure, interleukins as outcomes; or Causality of interleukins on OSA: interleukins

respectively as exposure and OSA as outcome. Each pair was analyzed by several MR methods. IVW: inverse variance weighted. MR RAPS: MR-Robust Adjusted Profile Score.

Supplemental Table 6. Verification of the causal effects of OSA on IL-6, IL-8 and IL-18 using another large-sample GWAS

Protein	Method	estimate	se	p-value	Q	Q_df	Q_pvalue	intercept	intercept_se	intercept_pvalue
IL-6	IVW	0.067	0.071	0.341	3.927	5	0.560	NA	NA	NA
	MR Egger	0.571	0.345	0.173	1.705	4	0.790	-0.045	0.030	0.210
	Weighted median	0.134	0.090	0.135	NA	NA	NA	NA	NA	NA
	MR RAPS	0.068	0.072	0.345	NA	NA	NA	NA	NA	NA
IL-8	IVW	-0.069	0.067	0.297	5.172	5	0.395	NA	NA	NA
	MR Egger	0.205	0.341	0.579	4.424	4	0.352	-0.025	0.030	0.457
	Weighted median	0.012	0.086	0.887	NA	NA	NA	NA	NA	NA
	MR RAPS	-0.071	0.067	0.289	NA	NA	NA	NA	NA	NA
IL-18	IVW	-0.043	0.061	0.488	0.413	5	0.995	NA	NA	NA
	MR Egger	-0.116	0.341	0.72	0.35	4	0.986	0.007	0.026	0.815
	Weighted median	-0.040	0.072	0.579	NA	NA	NA	NA	NA	NA
	MR RAPS	-0.042	0.063	0.503	NA	NA	NA	NA	NA	NA

Causality of OSA on interleukins : causality was analyzed with OSA as exposure, interleukins as outcomes; or Causality of interleukins on OSA: interleukins respectively as exposure and OSA as outcome. Each pair was analyzed by several MR methods. IVW: inverse variance weighted. MR RAPS: MR-Robust Adjusted Profile Score.

Supplemental Table 7. Causal effects of Interleukins (ILs) on OSA by Mendelian Randomization (MR) analysis.

Protein	Method	nsnp	estimate	se	p-value	Q	Q_df	Q_pvalue	intercept	intercept_se	intercept_pvalue
IL-1β	IVW	6	0.039273	0.071407	0.58233	4.729519	5	0.449773	NA	NA	NA
	MR Egger	6	0.010862	0.165456	0.950806	4.685345	4	0.321132	0.003512	0.018085	0.855486
	Weighted median	6	-0.01034	0.090972	0.909541	NA	NA	NA	NA	NA	NA
	MR RAPS	6	0.04114	0.077544	0.595737	NA	NA	NA	NA	NA	NA
IL-2	IVW	10	0.025366	0.030522	0.405922	8.744874	9	0.46115	NA	NA	NA
	MR Egger	10	-0.02266	0.062529	0.726473	7.970503	8	0.436357	0.009195	0.010449	0.404526
	Weighted median	10	0.015117	0.042427	0.721622	NA	NA	NA	NA	NA	NA
	MR RAPS	10	0.026348	0.032207	0.413315	NA	NA	NA	NA	NA	NA
IL-4	IVW	10	0.048121	0.049969	0.335534	8.623139	9	0.472763	NA	NA	NA
	MR Egger	10	0.038261	0.155176	0.811454	8.618242	8	0.375516	0.001188	0.017623	0.947902
	Weighted median	10	0.097065	0.069729	0.163913	NA	NA	NA	NA	NA	NA
	MR RAPS	10	0.049889	0.052471	0.341707	NA	NA	NA	NA	NA	NA
IL-5	IVW	6	0.04977	0.04044	0.218428	4.132199	5	0.530544	NA	NA	NA
	MR Egger	6	0.11575	0.098205	0.30386	3.588629	4	0.464531	-0.01202	0.016299	0.501866
	Weighted median	6	0.053002	0.051258	0.30113	NA	NA	NA	NA	NA	NA
	MR RAPS	6	0.051101	0.042606	0.230381	NA	NA	NA	NA	NA	NA
IL-6	IVW	7	0.049626	0.056975	0.383748	4.270568	6	0.640113	NA	NA	NA
	MR Egger	7	0.137867	0.114846	0.283733	3.487501	5	0.62528	-0.01274	0.0144	0.416724
	Weighted median	7	-0.0041	0.073238	0.955339	NA	NA	NA	NA	NA	NA
	MR RAPS	7	0.05088	0.060119	0.397368	NA	NA	NA	NA	NA	NA
IL-8	IVW	3	-0.04366	0.062591	0.485495	0.012108	2	0.993964	NA	NA	NA
	MR Egger	3	-0.0327	0.141117	0.855047	0.0046	1	0.945925	-0.00181	0.020926	0.944975
	Weighted median	3	-0.04219	0.073343	0.565098	NA	NA	NA	NA	NA	NA
	MR RAPS	3	-0.04366	0.067172	0.515668	NA	NA	NA	NA	NA	NA
IL-10	IVW	3	0.013154	0.038659	0.73367	0.322278	2	0.851174	NA	NA	NA
	MR Egger	3	0.016079	0.08902	0.886242	0.320948	1	0.571039	-0.00063	0.017151	0.976788
	Weighted median	3	0.013093	0.040712	0.747757	NA	NA	NA	NA	NA	NA
	MR RAPS	3	0.013164	0.039095	0.736329	NA	NA	NA	NA	NA	NA
IL-17	IVW	11	-0.02222	0.058493	0.704012	20.83394	10	0.022282	NA	NA	NA
	MR Egger	11	-0.10367	0.123623	0.423427	19.59996	9	0.020548	0.015001	0.019929	0.470833
	Weighted median	11	-0.04364	0.062549	0.485318	NA	NA	NA	NA	NA	NA
	MR RAPS	11	-0.02367	0.041822	0.571341	NA	NA	NA	NA	NA	NA
IL-18	IVW	12	-0.0137	0.024079	0.569385	12.45084	11	0.330726	NA	NA	NA
	MR Egger	12	-0.06025	0.058702	0.328903	11.57198	10	0.314723	0.010985	0.012605	0.403929
	Weighted median	12	-0.03063	0.02991	0.305738	NA	NA	NA	NA	NA	NA
	MR RAPS	12	-0.01404	0.023179	0.544612	NA	NA	NA	NA	NA	NA

Causality of interleukins on OSA : causality was analyzed with OSA as exposure, ILs as outcomes; or Causality of ILs on OSA: interleukins respectively as

exposure and OSA as outcome. Each pair was analyzed by several MR methods. IVW: inverse variance weighted. MR RAPS: MR-Robust Adjusted Profile Score.

Supplemental Table 8. Verification of the causal effects of IL-6, IL-8 and IL-18 on OSA using another large-sample GWAS

Protein	Method	estimate	se	p-value	Q	Q_df	Q_pvalue	intercept	intercept_se	intercept_pvalue
IL-6	IVW	-0.158	0.100	0.114	0.060	2	0.970	NA	NA	NA
	MR Egger	-0.447	2.503	0.888	0.0470	1	0.828	0.024	0.211	0.927
	Weighted median	-0.166	0.115	0.147	NA	NA	NA	NA	NA	NA
	MR RAPS	-0.063	0.061	0.308	NA	NA	NA	NA	NA	NA
IL-8	IVW	-0.018	0.163	0.912	5.074	2	0.079	NA	NA	NA
	MR Egger	0.802	0.482	0.344	1.247	1	0.264	-0.125	0.072	0.330
	Weighted median	0.033	0.134	0.804	NA	NA	NA	NA	NA	NA
	MR RAPS	-0.026	0.062	0.679	NA	NA	NA	NA	NA	NA
IL-18	IVW	-0.025	0.018	0.157	74.478	65	0.197	NA	NA	NA
	MR Egger	-0.051	0.049	0.300	74.112	64	0.182	0.004	0.006	0.576
	Weighted median	-0.031	0.026	0.233	NA	NA	NA	NA	NA	NA
	MR RAPS	-0.031	0.014	0.025	NA	NA	NA	NA	NA	NA

Causality of interleukins on OSA : causality was analyzed with OSA as exposure, ILs as outcomes; or Causality of ILs on OSA: interleukins respectively as exposure and OSA as outcome. Each pair was analyzed by several MR methods. IVW: inverse variance weighted. MR RAPS: MR-Robust Adjusted Profile Score.
