# Supplementary material for: Causal analysis between altered levels of interleukins and obstructive sleep apnea
Source: Front Immunol. 2022 Jul 27;13:888644. doi: 10.3389/fimmu.2022.888644 (PMC9363575; doi:10.3389/fimmu.2022.888644)
Supplement: Supplementary file 2 [file DataSheet_2.pdf]

Supplemental material 2: Supplemental Figures.

A. IL-1β

Study or Subgroup	Experimental Mean	Experimental SD	Experimental Total	Control Mean	Control SD	Control Total	Weight	Mean Difference IV, Random, 95% CI
Constantinidis J. 2008	0.28	0.11	13	0.66	0.21	12	10.5%	-0.38 [-0.51, -0.25]
Huang Y.S. 2016	0.36	0.16	47	0.42	0.27	32	10.5%	-0.06 [-0.16, 0.04]
Dalesio N.M. 2020	0.17	0.07	7	0.22	0.4	12	10.5%	-0.05 [-0.28, 0.18]
Sahlman J. 2010	0.19	1.51	84	0.23	1.31	40	10.3%	-0.04 [-0.56, 0.48]
Medeiros C.A.M. 2012	1.22	4.6	35	0	0	15		Not estimable
Tomiyama H. 2008	0.43	0.33	28	0.33	0.16	15	10.5%	0.10 [-0.05, 0.25]
Huang Y.S. 2020	1.51	2.25	55	0.42	1.38	32	10.1%	1.09 [0.33, 1.85]
Tang T. 2019	23	2.87	120	20.86	2.56	127	10.2%	2.14 [1.46, 2.82]
Chen V.G. 2020	102.03	112.98	17	97.26	139.67	17	0.0%	4.77 [-80.63, 90.17]
Kong Y. 2018	27.15	5.91	50	21.17	1.7	40	8.4%	5.98 [4.26, 7.70]
Chen B. 2021	13.17	5.5	23	2.67	1.89	30	7.2%	10.50 [8.15, 12.85]
Gamsiz-Isik H. 2017	38.82	26.42	83	19.25	17.65	80	2.1%	19.57 [12.70, 26.44]
Celikhisar H. 2020	23.74	2.98	30	1.7	0.21	82	9.6%	22.04 [20.97, 23.11]
**Total (95% CI)**			**592**			**534**	**100.0%**	**4.07 [2.96, 5.18]**

Heterogeneity: Tau² = 3.05; Chi² = 1872.19, df = 11 (P < 0.00001); I² = 99%
Test for overall effect: Z = 7.18 (P < 0.00001)

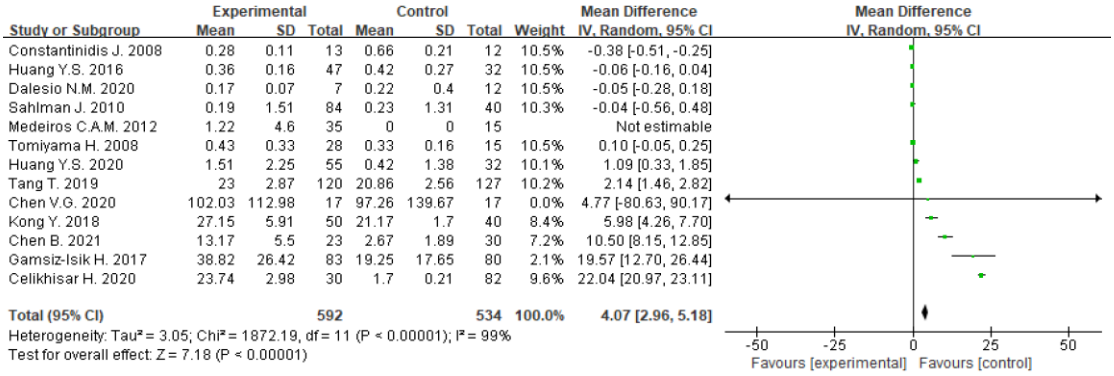

B. IL-2

Study or Subgroup	Experimental Mean	Experimental SD	Experimental Total	Control Mean	Control SD	Control Total	Weight	Mean Difference IV, Random, 95% CI
Said E.A. 2017	0.3	0.2	22	1.1	3.3	21	29.1%	-0.80 [-2.21, 0.61]
Zhang Z. 2017	3.41	0.21	50	3.38	0.19	52	30.0%	0.03 [-0.05, 0.11]
Sarinc Ulasli S. 2015	101.4	9.4	28	97.2	10.2	20	20.1%	4.20 [-1.47, 9.87]
Shi C. 2019	59.98	8.57	15	36.58	6.2	15	20.8%	23.40 [18.05, 28.75]
**Total (95% CI)**			**115**			**108**	**100.0%**	**5.49 [1.07, 9.92]**

Heterogeneity: Tau² = 16.97; Chi² = 76.62, df = 3 (P < 0.00001); I² = 96%
Test for overall effect: Z = 2.44 (P = 0.01)

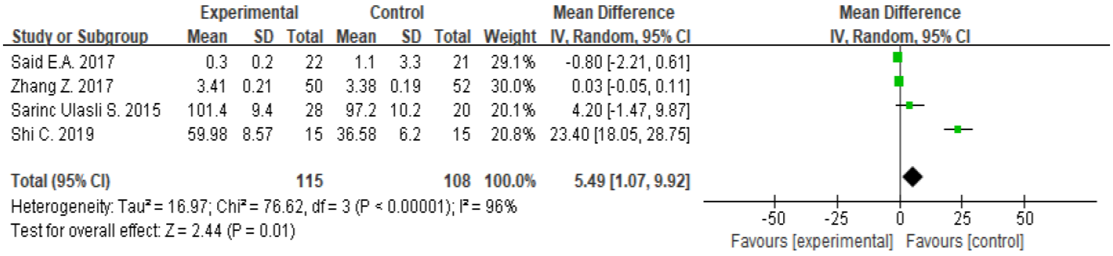

C. IL-4

Study or Subgroup	Experimental Mean	Experimental SD	Experimental Total	Control Mean	Control SD	Control Total	Weight	Mean Difference IV, Random, 95% CI
Said E.A. 2017	1.2	0.6	22	5.6	20.4	21	2.7%	-4.40 [-13.13, 4.33]
Akinnusi M. 2013	0.39	0.21	25	0.36	0.2	18	33.2%	0.03 [-0.09, 0.15]
Chen V.G. 2020	6.67	2.37	17	6.49	3.29	17	21.4%	0.18 [-1.75, 2.11]
Zhang Z. 2017	2.45	0.21	50	1.21	0.22	52	33.2%	1.24 [1.16, 1.32]
Ye J. 2015	36.72	16.58	25	33.24	15.69	19	2.3%	3.48 [-6.11, 13.07]
Su M. 2017	234.24	12.16	42	186.42	11.43	48	7.2%	47.82 [42.92, 52.72]
**Total (95% CI)**			**181**			**175**	**100.0%**	**3.89 [2.40, 5.38]**

Heterogeneity: Tau² = 1.74; Chi² = 607.83, df = 5 (P < 0.00001); I² = 99%
Test for overall effect: Z = 5.11 (P < 0.00001)

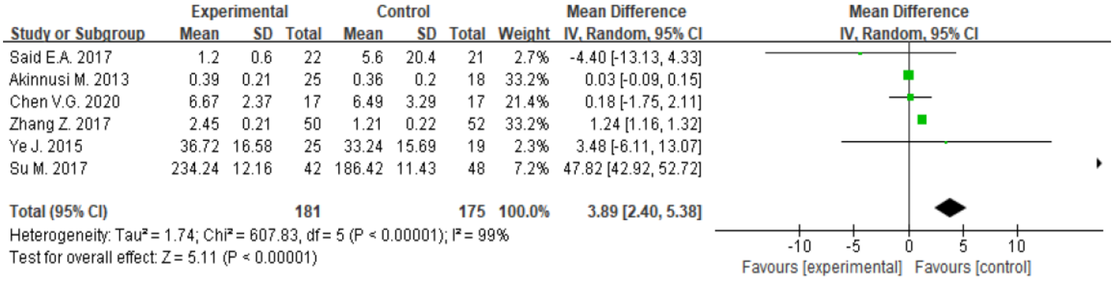

D. IL-6

Study or Subgroup	Experimental Mean	Experimental SD	Experimental Total	Control Mean	Control SD	Control Total	Weight	Mean Difference IV, Random, 95% CI
Roytblat L. 2000	5.58	0.37	11	7.69	5.06	9	1.1%	-2.11 [-5.42, 1.20]
Chen V.G. 2020	6.66	3.91	17	7.22	4.96	17	1.2%	-0.56 [-3.56, 2.44]
Kurt O.K. 2013	1.35	0.32	48	1.31	0.3	37	3.3%	0.04 [-0.09, 0.17]
Deboer M.D. 2012	1.69	1.1	9	1.61	1.27	15	2.8%	0.08 [-0.88, 1.04]
Zychowski K.E. 2016	2.23	0.04	8	2.15	0.05	7	3.3%	0.08 [0.03, 0.13]
Dalesio N.M. 2020	0.74	0.29	7	0.59	0.2	18	3.2%	0.15 [-0.08, 0.38]
Sahlman J. 2010	2.36	1.9	84	2.14	2.01	40	3.0%	0.22 [-0.52, 0.96]
Antonopoulou S. 2008	0.53	0.3	45	0.21	0.2	25	3.3%	0.32 [0.20, 0.44]
Akinnusi M. 2013	1.71	0.99	25	1.36	0.92	18	3.1%	0.35 [-0.23, 0.93]
Steiropoulos P. 2010	2.73	1.14	38	2.36	1.41	23	3.0%	0.37 [-0.31, 1.05]
Constantinidis J. 2008	0.84	0.08	13	0.42	0.08	12	3.3%	0.42 [0.36, 0.48]
Huang Y.S. 2020	1.59	1.58	55	1.1	0.92	32	3.1%	0.49 [-0.04, 1.02]
Nobili Y. 2015	11.5	5.9	52	11	5.6	28	1.5%	0.50 [-2.12, 3.12]
Kim J. 2010	3.57	2.74	28	3.02	2.44	22	2.4%	0.55 [-0.89, 1.99]
Huang Y.S. 2016	1.66	0.23	47	1.1	0.18	32	3.3%	0.56 [0.47, 0.65]
Takahashi K.I. 2008	1.56	2.49	41	0.99	0.94	12	2.8%	0.57 [-0.36, 1.50]
Arias M.A. 2008	4.2	3.4	30	3.6	1.8	15	2.3%	0.60 [-0.92, 2.12]
Liu Z. 2011	1.62	0.43	20	0.92	0.34	20	3.2%	0.70 [0.46, 0.94]
Gileles-Hillel A. 2014	8	4.8	75	7.3	3.2	129	2.6%	0.70 [-0.52, 1.92]
Qian X. 2012	89.56	36.19	30	88.85	41.48	40	0.1%	0.71 [-17.54, 18.96]
Yang D. 2013	1.89	1.29	25	1	0.12	25	3.1%	0.89 [0.38, 1.40]
Tomiyama H. 2008	2.4	1.6	28	1.5	0.6	15	3.0%	0.90 [0.23, 1.57]
Nakra N. 2008	3.9	4.3	24	3	2.4	9	1.6%	0.90 [-1.43, 3.23]
Mônico-Neto M. 2018	10.1	0.8	123	9.1	0.9	211	3.3%	1.00 [0.81, 1.19]
Dogan D. 2016	2.02	1.19	39	1.01	0.19	12	3.2%	1.01 [0.62, 1.40]
Kotsiou O. S. 2022	3.4	1	15	2.3	0.3	15	3.1%	1.10 [0.57, 1.63]
Tauman R. 2007	2.26	2.7	38	1.12	0.7	33	2.9%	1.14 [0.25, 2.03]
Zhang Z. 2017	3.75	0.19	50	2.54	0.17	52	3.3%	1.21 [1.14, 1.28]
Damiani M.F. 2015	3.14	1.27	30	1.46	0.69	30	3.1%	1.68 [1.16, 2.20]
Shalitin S. 2018	4.56	2.92	9	2.83	1.54	29	1.9%	1.73 [-0.26, 3.72]
Ciccone M.M. 2014	3.33	1.35	54	1.5	0.67	40	3.2%	1.83 [1.41, 2.25]
Bozic J. 2018	3.58	1.55	25	1.26	0.69	25	3.0%	2.32 [1.65, 2.99]
Ifergane G. 2016	6.64	5.27	21	3.14	2.05	22	1.6%	3.50 [1.09, 5.91]
Tang T. 2019	63.6	8.29	120	59.16	12.27	127	1.5%	4.44 [1.84, 7.04]
Smith D.F. 2017	29.2	62	42	24.6	51.9	90	0.0%	4.60 [-17.00, 26.20]
Bhatt S.P. 2021	20.1	5.6	190	15.4	3.5	57	2.6%	4.70 [3.49, 5.91]
Ciftci T.U. 2004	11.71	9.8	43	6.12	4.44	22	1.0%	5.59 [2.12, 9.06]
Kong Y. 2018	61.59	9.76	50	54.46	9.43	40	0.8%	7.13 [3.15, 11.11]
Medeiros C.A.M. 2012	9.4	13.2	35	0.77	2.53	15	0.7%	8.63 [4.07, 13.19]
Ye J. 2012	55.09	17.02	44	42.56	21.15	20	0.2%	12.53 [1.98, 23.08]
Nizam N. 2016	99.2	41.1	22	82.4	27.2	13	0.0%	16.80 [-5.86, 39.46]
Bilal N. 2021	57.98	5.96	10	40.57	3.47	30	0.9%	17.41 [13.51, 21.31]
Li Y 2008	65.3	15.4	28	37.5	10.9	22	0.3%	27.80 [20.50, 35.10]
Chen J. 2007	143.12	33.52	100	107.43	27.66	40	0.1%	35.69 [24.89, 46.49]
Liu H. 2000	50.67	4.7	22	12.69	2.75	16	1.6%	37.98 [35.60, 40.36]
Li Y 2009	83.4	6.4	24	37.5	10.9	22	0.5%	45.90 [40.67, 51.13]
Lu D. 2018	58.49	8.03	35	9.11	10.13	22	0.6%	49.38 [44.38, 54.38]
Ye L. 2010	144.5	10.9	45	53.6	3.5	52	1.1%	90.90 [87.58, 94.22]
**Total (95% CI)**			**1974**			**1657**	**100.0%**	**3.49 [3.07, 3.92]**

Heterogeneity: $Tau^2 = 1.43$; $Chi^2 = 5635.81$, df = 47 ($P < 0.00001$); $I^2 = 99\%$
Test for overall effect: $Z = 16.13$ ($P < 0.00001$)

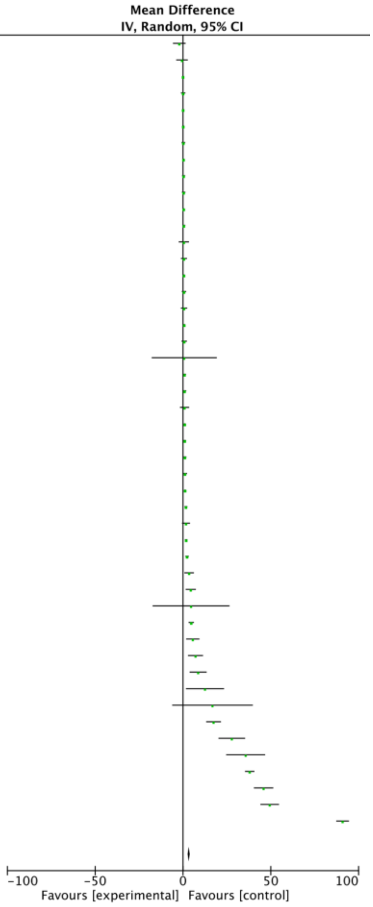

E. IL-8

Study or Subgroup	Experimental Mean	Experimental SD	Experimental Total	Control Mean	Control SD	Control Total	Weight	Mean Difference IV, Random, 95% CI
Said E.A. 2017	75.2	32.9	22	91.5	43.5	21	5.4%	-16.30 [-39.43, 6.83]
Archontogeorgis K. 2016	31.79	20.09	64	34.09	14.37	20	8.8%	-2.30 [-10.29, 5.69]
Cheng J. 2021	43.5	7.22	29	45.65	6.87	22	9.4%	-2.15 [-6.04, 1.74]
Akinnusi M. 2013	4.98	1.67	25	4.57	1.453	18	9.6%	0.41 [-0.53, 1.35]
Kim J. 2010	13.35	5.63	28	12.89	4.3	22	9.5%	0.46 [-2.29, 3.21]
Smith D.F. 2017	11.6	16.7	42	9.8	18.8	90	9.1%	1.80 [-4.57, 8.17]
Bhatt S.P. 2021	8.29	5.2	190	4.98	3.67	57	9.6%	3.31 [2.10, 4.52]
Bao H.R. 2005	35.95	10.63	35	22.27	7.62	25	9.3%	13.68 [9.06, 18.30]
Ming H. 2019	34.6	7.1	684	19.4	8.5	192	9.6%	15.20 [13.89, 16.51]
Bilal N. 2021	126.31	11.73	10	100.12	5.13	30	8.9%	26.19 [18.69, 33.69]
Jin F. 2017	40.72	1.6	100	13.28	3.15	50	9.6%	27.44 [26.51, 28.37]
Zhang D. 2019	229.71	175.61	21	69.4	26.7	10	1.0%	160.31 [83.40, 237.22]
Chen V.G. 2020	1,088.77	1,012.05	17	664.44	565.13	17	0.0%	424.33 [-126.68, 975.34]
**Total (95% CI)**			**1267**			**574**	**100.0%**	**8.68 [0.56, 16.80]**

Heterogeneity: $Tau^2 = 178.16$; $Chi^2 = 2046.15$, df = 12 ($P < 0.00001$); $I^2 = 99\%$
Test for overall effect: $Z = 2.10$ ($P = 0.04$)

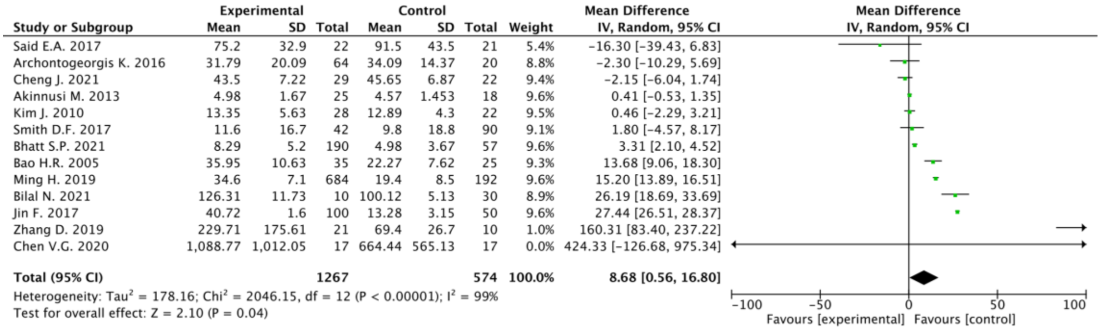

F. IL-10

Study or Subgroup	Experimental Mean	Experimental SD	Experimental Total	Control Mean	Control SD	Control Total	Weight	Mean Difference IV, Random, 95% CI
Su M. 2017	261.71	18.55	42	338.24	25.2	48	0.3%	-76.53 [-85.60, -67.46]
Li Y 2009	24.6	5.1	24	50.1	6.9	22	1.7%	-25.50 [-29.03, -21.97]
Leon-Cabrera S. 2015	74.4	17	29	97.2	10.9	13	0.3%	-22.80 [-31.37, -14.23]
Li Y 2008	37.5	12.4	28	50.1	6.9	22	0.8%	-12.60 [-18.02, -7.18]
Bao H.R. 2005	36.39	4.96	35	42.06	4.09	25	3.5%	-5.67 [-7.97, -3.37]
Ye J. 2012	47.84	10.72	44	52.17	23.18	20	0.2%	-4.33 [-14.97, 6.31]
Said E.A. 2017	0.5	0.3	22	1.8	5.9	21	3.0%	-1.30 [-3.83, 1.23]
Rogers V.E. 2018	1.7	1.06	20	2.03	1.76	7	6.5%	-0.33 [-1.71, 1.05]
Akinnusi M. 2013	0.96	0.48	25	0.89	0.43	18	12.4%	0.07 [-0.20, 0.34]
Dalesio N.M. 2020	0.59	0.17	7	0.46	0.16	18	12.7%	0.13 [-0.02, 0.28]
Zhang Z. 2017	3.45	0.19	50	3.01	0.17	52	12.8%	0.44 [0.37, 0.51]
Bhatt S.P. 2021	2.62	0.39	190	2.1	0.28	57	12.8%	0.52 [0.43, 0.61]
Huang Y.S. 2016	2.62	0.39	47	2.1	0.28	32	12.7%	0.52 [0.37, 0.67]
Sahlman J. 2010	1.28	2.34	84	0.7	1.51	40	10.4%	0.58 [-0.11, 1.27]
Huang Y.S. 2020	2.74	2.94	55	2.1	1.41	32	9.0%	0.64 [-0.28, 1.56]
Sarinc Ulasli S. 2015	34.7	12.4	28	32.5	14	20	0.4%	2.20 [-5.46, 9.86]
Chen V.G. 2020	13.08	11.77	17	10.69	12.95	17	0.4%	2.39 [-5.93, 10.71]
**Total (95% CI)**			**747**			**464**	**100.0%**	**-0.77 [-1.27, -0.27]**

Heterogeneity: Tau² = 0.51; Chi² = 593.23, df = 16 (P < 0.00001); I² = 97%
Test for overall effect: Z = 3.00 (P = 0.003)

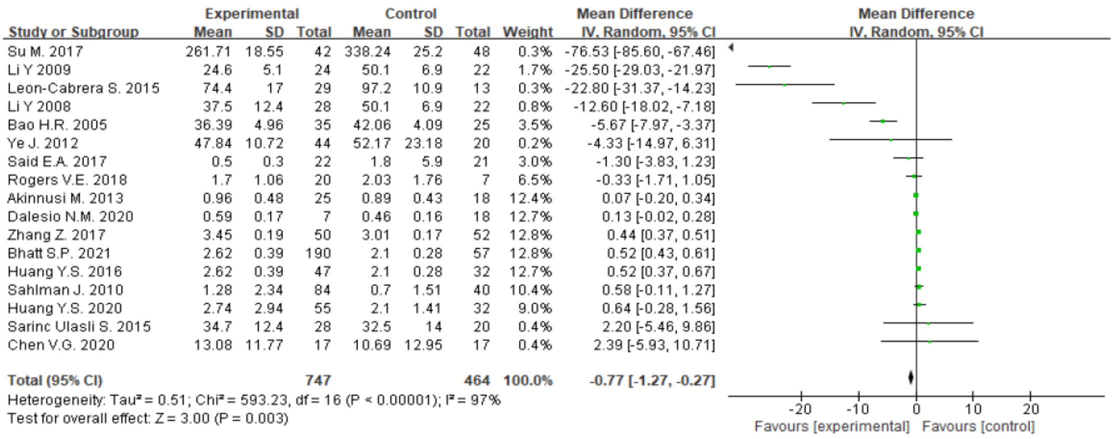

G. IL-12

Study or Subgroup	Experimental Mean	Experimental SD	Experimental Total	Control Mean	Control SD	Control Total	Weight	Mean Difference IV, Fixed, 95% CI
Rogers V.E. 2018	4.88	2.97	20	5.37	4.15	7	2.9%	-0.49 [-3.83, 2.85]
Huang Y.S. 2020	1.77	1.15	55	0.97	0.75	6	70.2%	0.80 [0.13, 1.47]
Said E.A. 2017	1	2.6	22	0	0.1	21	26.9%	1.00 [-0.09, 2.09]
Leon-Cabrera S. 2015	405.3	57.4	29	394	64.7	13	0.0%	11.30 [-29.61, 52.21]
**Total (95% CI)**			**126**			**47**	**100.0%**	**0.82 [0.26, 1.38]**

Heterogeneity: Chi² = 0.95, df = 3 (P = 0.81); I² = 0%
Test for overall effect: Z = 2.85 (P = 0.004)

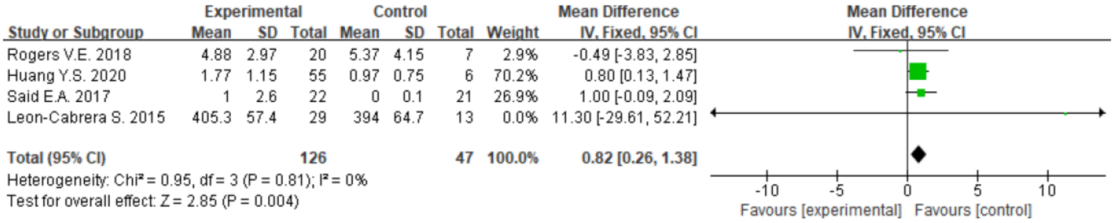

H. IL-17

Study or Subgroup	Experimental Mean	Experimental SD	Experimental Total	Control Mean	Control SD	Control Total	Weight	Mean Difference IV, Random, 95% CI
Bhatt S.P. 2021	15.12	1.38	190	10.2	1.25	57	24.8%	4.92 [4.54, 5.30]
Huang Y.S. 2016	15.12	1.38	47	10.2	1.25	32	24.5%	4.92 [4.33, 5.51]
Huang Y.S. 2020	13.78	7.18	55	10.2	6.36	32	18.2%	3.58 [0.67, 6.49]
Toujani S. 2017	20.3	3.9	92	10.05	3	30	23.1%	10.25 [8.91, 11.59]
Ye J. 2012	70.3	13.65	44	55.12	18.23	20	5.6%	15.18 [6.23, 24.13]
Ye J. 2015	88.36	14.96	25	38.18	21.63	19	3.8%	50.18 [38.82, 61.54]
**Total (95% CI)**			**453**			**190**	**100.0%**	**8.19 [5.79, 10.59]**

Heterogeneity: Tau² = 6.01; Chi² = 123.96, df = 5 (P < 0.00001); I² = 96%
Test for overall effect: Z = 6.70 (P < 0.00001)

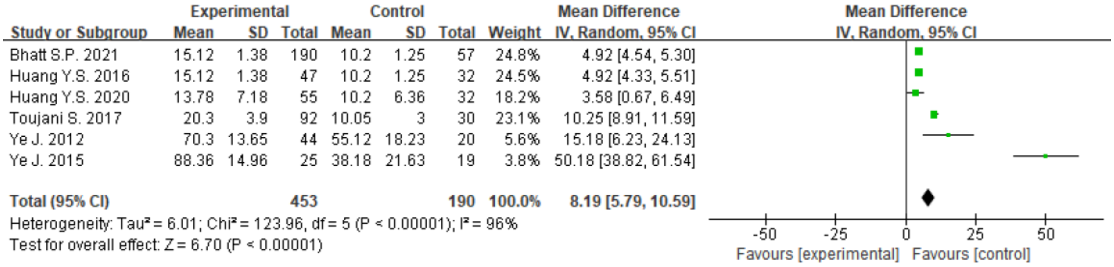

I. IL-18

Study or Subgroup	Experimental Mean	Experimental SD	Experimental Total	Control Mean	Control SD	Control Total	Weight	Mean Difference IV, Random, 95% CI
Tang T. 2019	94.13	7.98	120	89.72	12.71	127	41.4%	4.41 [1.78, 7.04]
Zhang S.W. 2015	19.26	9.87	34	10.58	2.02	25	41.3%	8.68 [5.27, 12.09]
Li C 2009	797.64	109.83	18	250.27	76.48	18	17.3%	547.37 [485.54, 609.20]
**Total (95% CI)**			**172**			**170**	**100.0%**	**100.14 [66.45, 133.84]**

Heterogeneity: Tau² = 712.26; Chi² = 297.96, df = 2 (P < 0.00001); I² = 99%
Test for overall effect: Z = 5.83 (P < 0.00001)

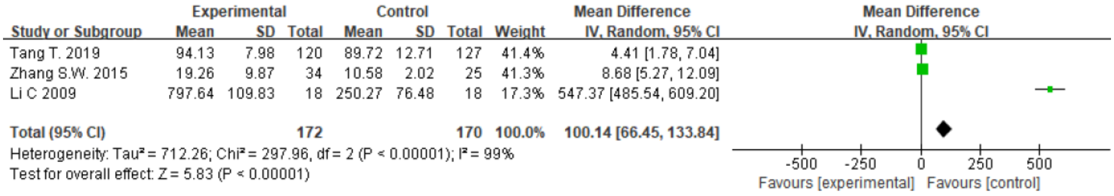

J. IL-23

Study or Subgroup	Experimental Mean	Experimental SD	Experimental Total	Control Mean	Control SD	Control Total	Weight	Mean Difference IV, Random, 95% CI
Huang Y.S. 2016	14.58	0.75	47	12.29	0.73	32	25.3%	2.29 [1.96, 2.62]
Bhatt S.P. 2021	14.58	0.75	190	12.29	0.73	57	25.3%	2.29 [2.07, 2.51]
Huang Y.S. 2020	20.14	7.08	55	12.29	3.66	32	24.4%	7.85 [5.59, 10.11]
Can M. 2016	30.9	1.4	12	6.9	1.3	27	25.1%	24.00 [23.07, 24.93]
**Total (95% CI)**			**304**			**148**	**100.0%**	**9.10 [3.21, 14.98]**

Heterogeneity: Tau² = 35.66; Chi² = 2026.58, df = 3 (P < 0.00001); I² = 100%
Test for overall effect: Z = 3.03 (P = 0.002)

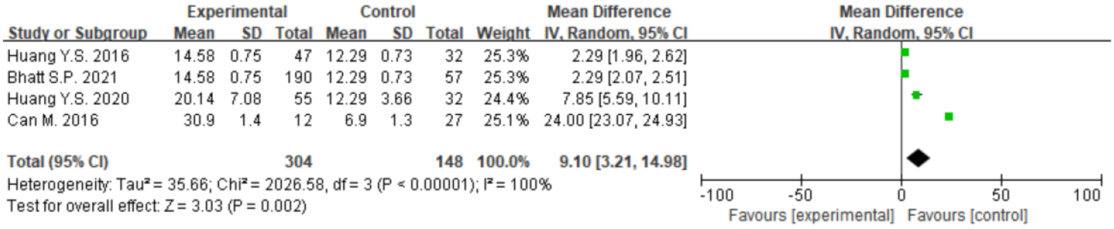

Supplemental Figure 1. Forest plots of interleukins (ILs) concentration differences between OSA patients and controls. (A) IL-1β; (B) IL-2; (C) IL-4; (D) IL-6; (E) IL-8; (F) IL-10; (G) IL-12; (H) IL-17; (I) IL-18; (J) IL-23.

The study of Medeiros C.A.M made no contribution to the pooled analysis in IL-1β.

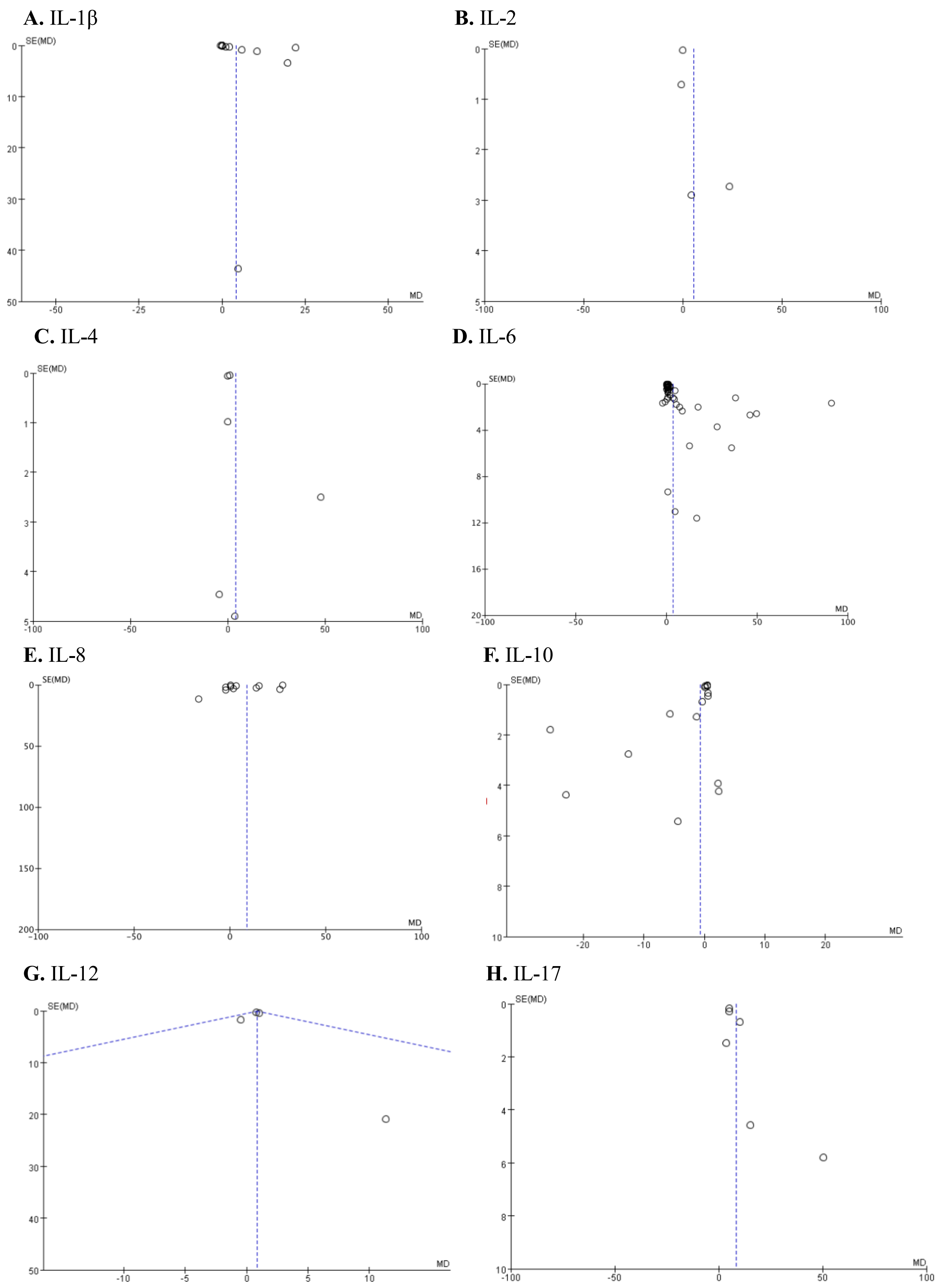

A. IL-1β
SE(MD)
MD
B. IL-2
SE(MD)
MD
C. IL-4
SE(MD)
MD
D. IL-6
SE(MD)
MD
E. IL-8
SE(MD)
MD
F. IL-10
SE(MD)
MD
G. IL-12
SE(MD)
MD
H. IL-17
SE(MD)
MD

I. IL-18

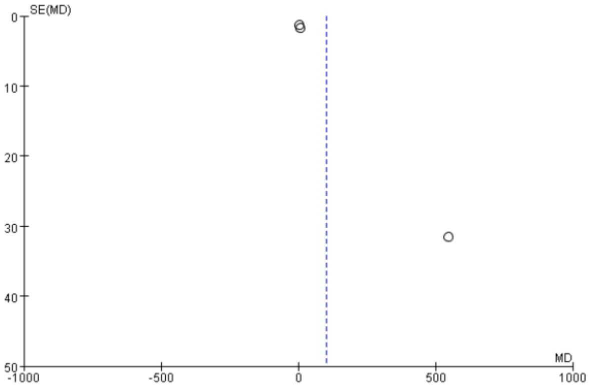

J. IL-23

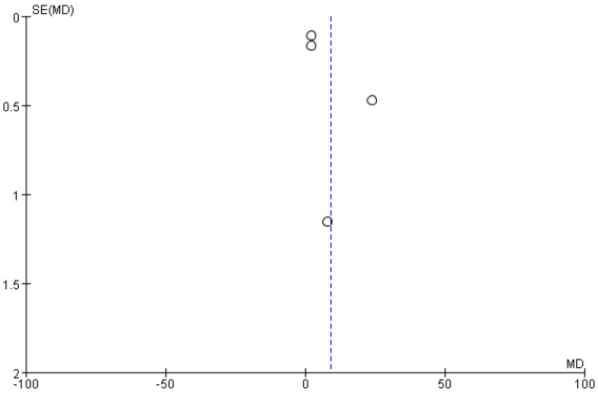

Supplemental Figure 2. Funnel plots of Interleukins (ILs) concentration differences between OSA patients and controls. (A) IL-1β; (B) IL-2; (C) IL-4; (D) IL-6; (E) IL-8; (F) IL-10; (G) IL-12; (H) IL-17; (I) IL-18; (J) IL-23.

A. IL-1β

Study or Subgroup	Experimental Mean	Experimental SD	Experimental Total	Control Mean	Control SD	Control Total	Weight	Mean Difference IV, Random, 95% CI
**2.3.1 mild OSA VS no OSA**								
Kong Y. 2018	20.1	1.47	10	21.17	1.7	40	6.0%	-1.07 [-2.12, -0.02]
Tang T. 2019	20.1	1.48	9	20.86	2.56	127	6.0%	-0.76 [-1.82, 0.30]
Sahlman J. 2010	0.19	1.51	84	0.23	1.31	40	6.3%	-0.04 [-0.56, 0.48]
Medeiros C.A.M. 2012	0	0	15	0	0	15		Not estimable
Tomiyama H. 2008	0.35	0.12	10	0.33	0.16	15	6.4%	0.02 [-0.09, 0.13]
Chen B. 2021	7.29	3.43	20	2.67	1.89	30	5.5%	4.62 [2.97, 6.27]
Celikhisar H. 2020	11.8	7.04	28	1.7	0.21	82	4.6%	10.10 [7.49, 12.71]
Gamsiz-Isik H. 2017	34.35	24.2	16	19.25	17.65	80	0.6%	15.10 [2.63, 27.57]
**Subtotal (95% CI)**			**192**			**429**	**35.6%**	**1.59 [0.33, 2.86]**
Heterogeneity: Tau² = 2.11; Chi² = 99.07, df = 6 (P < 0.00001); I² = 94%								
Test for overall effect: Z = 2.47 (P = 0.01)								
**2.3.2 moderate OSA VS no OSA**								
Tomiyama H. 2008	0.4	0.28	12	0.33	0.16	15	6.4%	0.07 [-0.11, 0.25]
Tang T. 2019	22.89	1.92	33	20.86	2.56	127	6.2%	2.03 [1.24, 2.82]
Kong Y. 2018	24.35	3.83	15	21.17	1.7	40	5.2%	3.18 [1.17, 5.19]
Chen B. 2021	10.69	3.83	24	2.67	1.89	30	5.5%	8.02 [6.35, 9.69]
Celikhisar H. 2020	17.2	5.96	26	1.7	0.21	82	4.9%	15.50 [13.21, 17.79]
Gamsiz-Isik H. 2017	39.82	26.92	67	19.25	17.65	80	1.4%	20.57 [13.05, 28.09]
**Subtotal (95% CI)**			**177**			**374**	**29.7%**	**7.14 [3.61, 10.68]**
Heterogeneity: Tau² = 17.45; Chi² = 310.68, df = 5 (P < 0.00001); I² = 98%								
Test for overall effect: Z = 3.96 (P < 0.0001)								
**2.3.3 severe OSA VS no OSA**								
Dalesio N.M. 2020	0.17	0.07	7	0.22	0.4	12	6.4%	-0.05 [-0.28, 0.18]
Medeiros C.A.M. 2012	1.22	4.6	35	0	0	15		Not estimable
Tomiyama H. 2008	0.43	0.33	28	0.33	0.16	15	6.4%	0.10 [-0.05, 0.25]
Tang T. 2019	24.92	2.86	78	20.86	2.56	127	6.2%	4.06 [3.28, 4.84]
Kong Y. 2018	29.15	5.91	25	21.17	1.7	40	4.8%	7.98 [5.60, 10.36]
Chen B. 2021	13.17	5.5	23	2.67	1.89	30	4.8%	10.50 [8.15, 12.85]
Celikhisar H. 2020	23.74	2.98	30	1.7	0.21	82	6.0%	22.04 [20.97, 23.11]
**Subtotal (95% CI)**			**226**			**321**	**34.8%**	**7.34 [4.18, 10.51]**
Heterogeneity: Tau² = 15.09; Chi² = 1803.46, df = 5 (P < 0.00001); I² = 100%								
Test for overall effect: Z = 4.55 (P < 0.00001)								
**Total (95% CI)**			**595**			**1124**	**100.0%**	**4.96 [3.94, 5.99]**
Heterogeneity: Tau² = 4.25; Chi² = 2245.35, df = 18 (P < 0.00001); I² = 99%								
Test for overall effect: Z = 9.47 (P < 0.00001)								
Test for subgroup differences: Chi² = 17.19, df = 2 (P = 0.0002), I² = 88.4%								

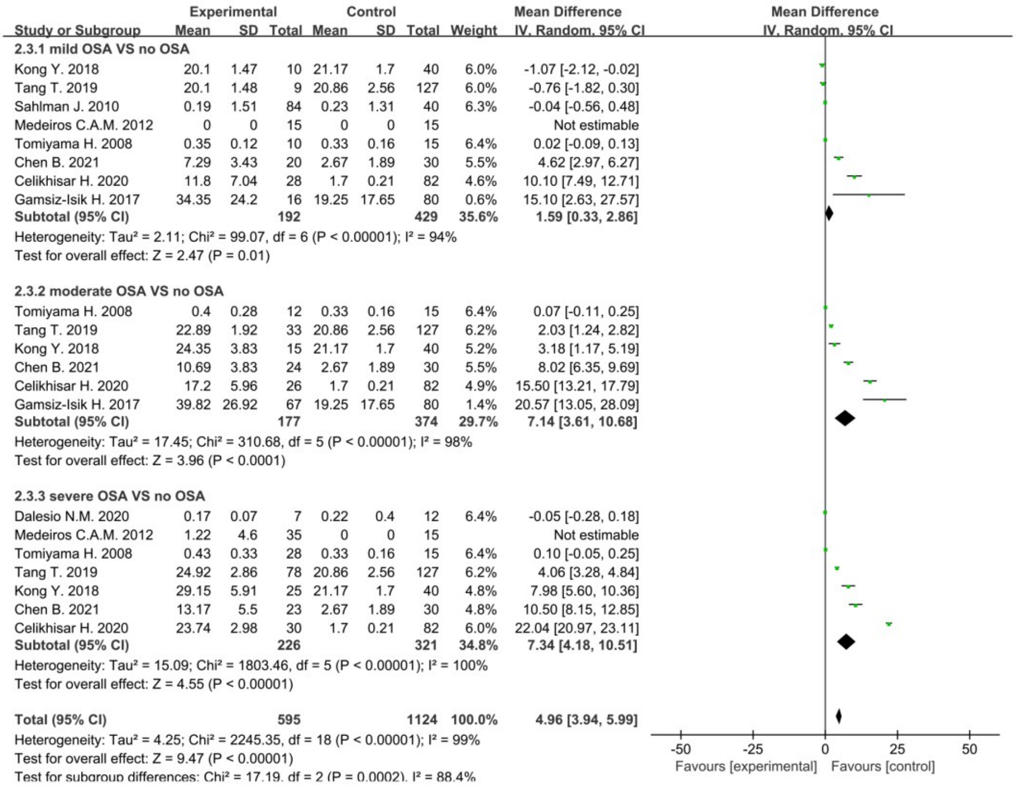

B. IL-6

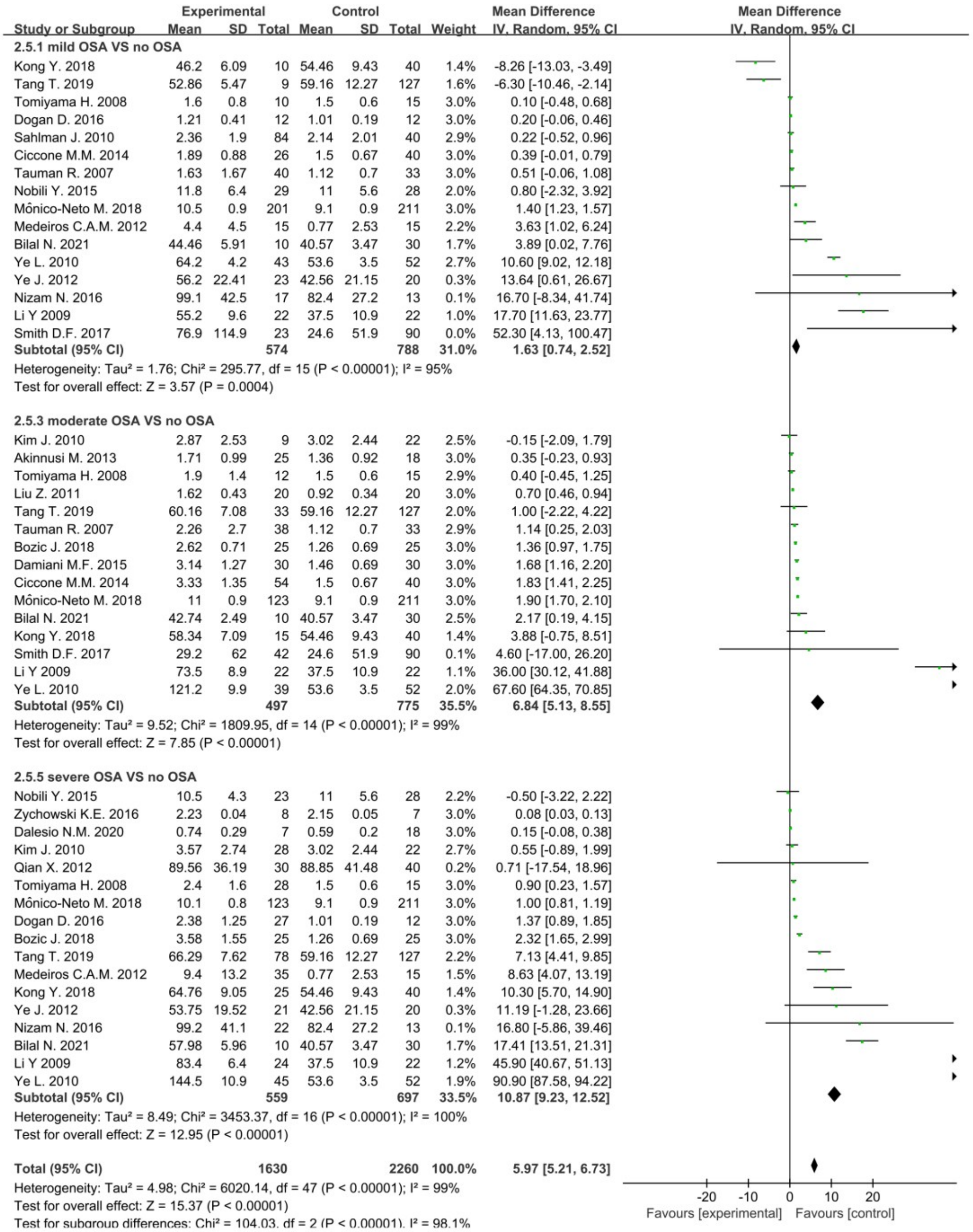

Supplemental Figure 3. Forest plots of IL-1β for IL-6 concentration differences between mild, moderate, severe OSA patients and controls. (A) IL-1β; (B) IL-6.

The study of Medeiros C.A.M made no contribution to the pooled analysis in IL-1β.

A. IL-8

Study or Subgroup	Experimental Mean	Experimental SD	Experimental Total	Control Mean	Control SD	Control Total	Weight	Mean Difference IV, Random, 95% CI
**2.6.1 moderate OSA VS no OSA**								
Kim J. 2010	11.9	2.65	9	12.89	4.3	22	19.1%	−0.99 [−3.49, 1.51]
Akinnusi M. 2013	4.98	1.67	25	4.57	1.453	18	20.5%	0.41 [−0.53, 1.35]
Smith D.F. 2017	11.6	16.7	42	9.8	18.8	90	13.4%	1.80 [−4.57, 8.17]
Bilal N. 2021	105.66	9.22	10	100.12	5.13	30	13.9%	5.54 [−0.46, 11.54]
**Subtotal (95% CI)**			**86**			**160**	**66.9%**	**0.45 [−1.14, 2.03]**
Heterogeneity: Tau² = 0.83; Chi² = 4.19, df = 3 (P = 0.24); I² = 28%								
Test for overall effect: Z = 0.55 (P = 0.58)								
**2.6.2 severe OSA VS no OSA**								
Said E.A. 2017	75.2	32.9	22	91.5	43.5	21	2.5%	−16.30 [−39.43, 6.83]
Kim J. 2010	13.35	5.63	28	12.89	4.3	22	18.8%	0.46 [−2.29, 3.21]
Bilal N. 2021	126.31	11.73	10	100.12	5.13	30	11.8%	26.19 [18.69, 33.69]
**Subtotal (95% CI)**			**60**			**73**	**33.1%**	**5.41 [−15.69, 26.51]**
Heterogeneity: Tau² = 305.33; Chi² = 42.64, df = 2 (P < 0.00001); I² = 95%								
Test for overall effect: Z = 0.50 (P = 0.62)								
**Total (95% CI)**			**146**			**233**	**100.0%**	**3.67 [−0.25, 7.58]**
Heterogeneity: Tau² = 19.28; Chi² = 51.23, df = 6 (P < 0.00001); I² = 88%								
Test for overall effect: Z = 1.84 (P = 0.07)								
Test for subgroup differences: Chi² = 0.21, df = 1 (P = 0.65), I² = 0%								

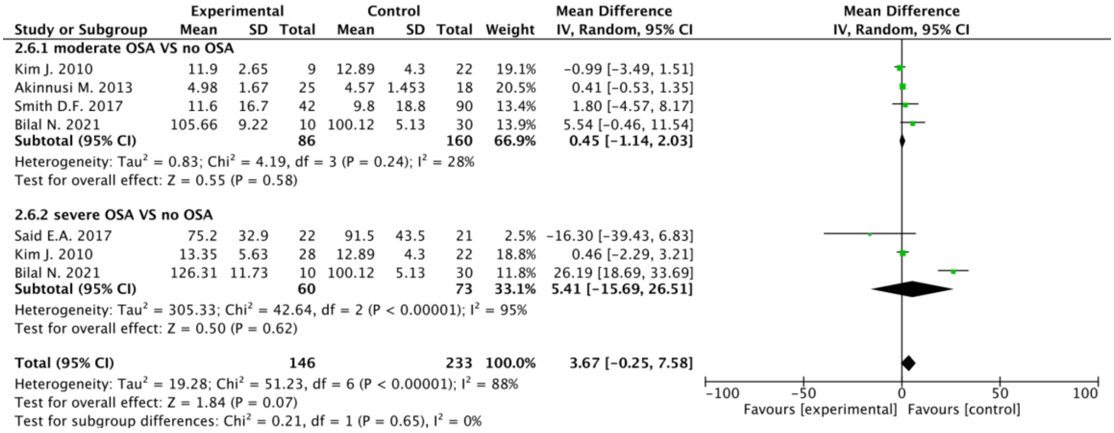

B. IL-10

Study or Subgroup	Experimental Mean	Experimental SD	Experimental Total	Control Mean	Control SD	Control Total	Weight	Mean Difference IV, Random, 95% CI
**2.7.1 mild OSA VS no OSA**								
Ye J. 2012	46.79	11.69	23	52.17	23.18	20	5.2%	-5.38 [-16.61, 5.85]
Li Y 2009	46.7	4.6	22	50.1	6.9	22	15.8%	-3.40 [-6.87, 0.07]
Sahlman J. 2010	1.28	2.34	84	0.7	1.51	40	20.0%	0.58 [-0.11, 1.27]
**Subtotal (95% CI)**			**129**			**82**	**41.0%**	**-1.38 [-4.91, 2.16]**
Heterogeneity: Tau² = 5.79; Chi² = 5.90, df = 2 (P = 0.05); I² = 66%								
Test for overall effect: Z = 0.76 (P = 0.45)								
**2.7.3 severe OSA VS no OSA**								
Li Y 2009	24.6	5.1	24	50.1	6.9	22	15.7%	-25.50 [-29.03, -21.97]
Ye J. 2012	49.06	8.51	21	52.17	23.18	20	5.5%	-3.11 [-13.90, 7.68]
Said E.A. 2017	0.5	0.3	22	1.8	5.9	21	17.6%	-1.30 [-3.83, 1.23]
Dalesio N.M. 2020	0.59	0.17	7	0.46	0.16	18	20.2%	0.13 [-0.02, 0.28]
**Subtotal (95% CI)**			**74**			**81**	**59.0%**	**-7.58 [-18.06, 2.89]**
Heterogeneity: Tau² = 106.71; Chi² = 203.40, df = 3 (P < 0.00001); I² = 99%								
Test for overall effect: Z = 1.42 (P = 0.16)								
**Total (95% CI)**			**203**			**163**	**100.0%**	**-5.08 [-8.03, -2.12]**
Heterogeneity: Tau² = 11.27; Chi² = 210.18, df = 6 (P < 0.00001); I² = 97%								
Test for overall effect: Z = 3.36 (P = 0.0008)								
Test for subgroup differences: Chi² = 1.21, df = 1 (P = 0.27), I² = 17.4%								

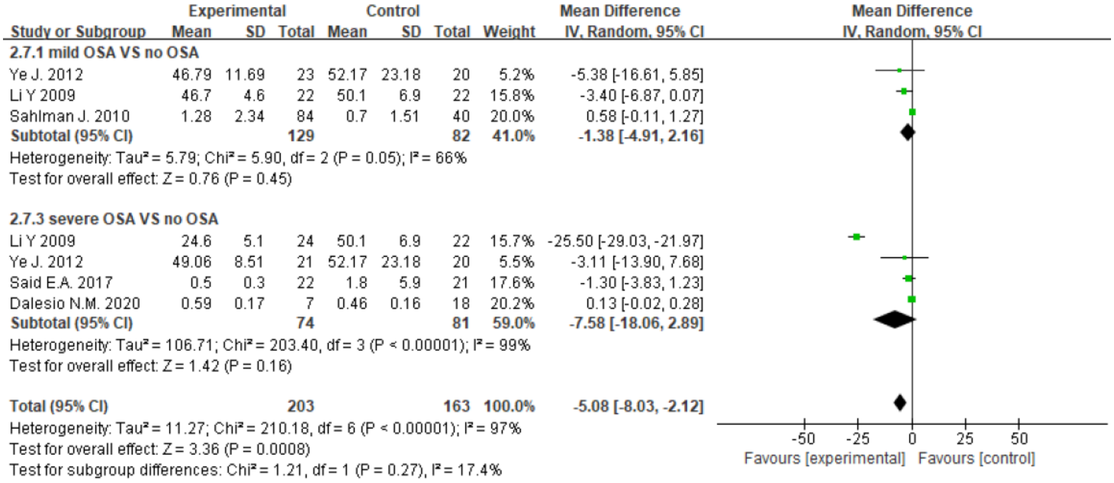

C. IL-18

Study or Subgroup	Experimental Mean	Experimental SD	Experimental Total	Control Mean	Control SD	Control Total	Weight	Mean Difference IV, Random, 95% CI
**2.11.1 mild OSA VS no OSA**								
Li C 2009	352.08	76.32	16	250.27	76.48	18	5.8%	101.81 [50.36, 153.26]
Tang T. 2019	81.8	2.68	9	89.72	12.71	127	22.5%	-7.92 [-10.74, -5.10]
Zhang S.W. 2015	15.57	5.09	32	10.58	2.02	25	22.6%	4.99 [3.06, 6.92]
**Subtotal (95% CI)**			**57**			**170**	**50.8%**	**4.93 [-8.76, 18.62]**
Heterogeneity: Tau² = 102.42; Chi² = 69.55, df = 2 (P < 0.00001); I² = 97%								
Test for overall effect: Z = 0.71 (P = 0.48)								
**2.11.3 severe OSA VS no OSA**								
Li C 2009	797.64	109.83	18	250.27	76.48	18	4.3%	547.37 [485.54, 609.20]
Tang T. 2019	96.77	7.63	78	89.72	12.71	127	22.5%	7.05 [4.27, 9.83]
Zhang S.W. 2015	19.26	9.87	34	10.58	2.02	25	22.4%	8.68 [5.27, 12.09]
**Subtotal (95% CI)**			**130**			**170**	**49.2%**	**102.42 [68.33, 136.51]**
Heterogeneity: Tau² = 731.11; Chi² = 292.85, df = 2 (P < 0.00001); I² = 99%								
Test for overall effect: Z = 5.89 (P < 0.00001)								
**Total (95% CI)**			**187**			**340**	**100.0%**	**32.57 [18.22, 46.91]**
Heterogeneity: Tau² = 236.33; Chi² = 391.65, df = 5 (P < 0.00001); I² = 99%								
Test for overall effect: Z = 4.45 (P < 0.00001)								
Test for subgroup differences: Chi² = 27.05, df = 1 (P < 0.00001), I² = 96.3%								

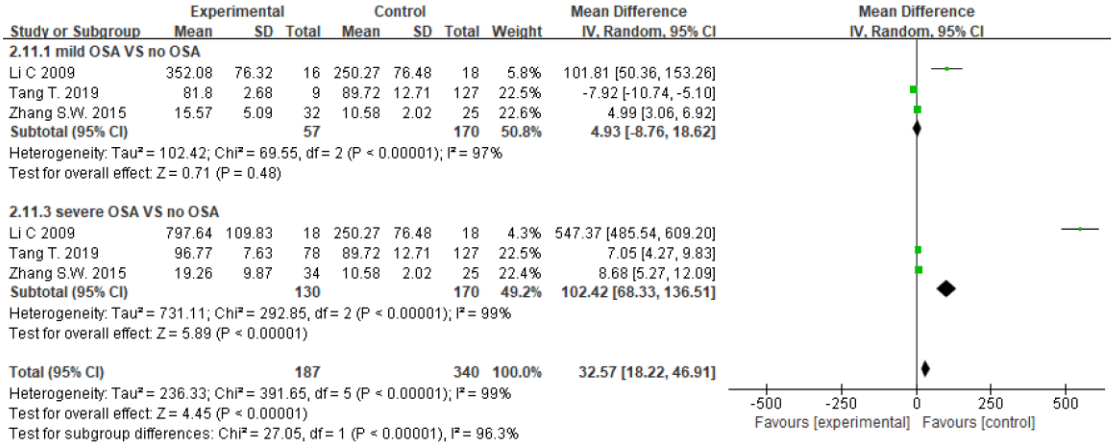

Supplemental Figure 4. Forest plots of interleukins (ILs) concentration differences between 2 subgroups of OSA patients and controls. (A) IL-8 in moderate, severe subgroups; (B) and (C) IL-10, IL-18 in mild, severe subgroups.

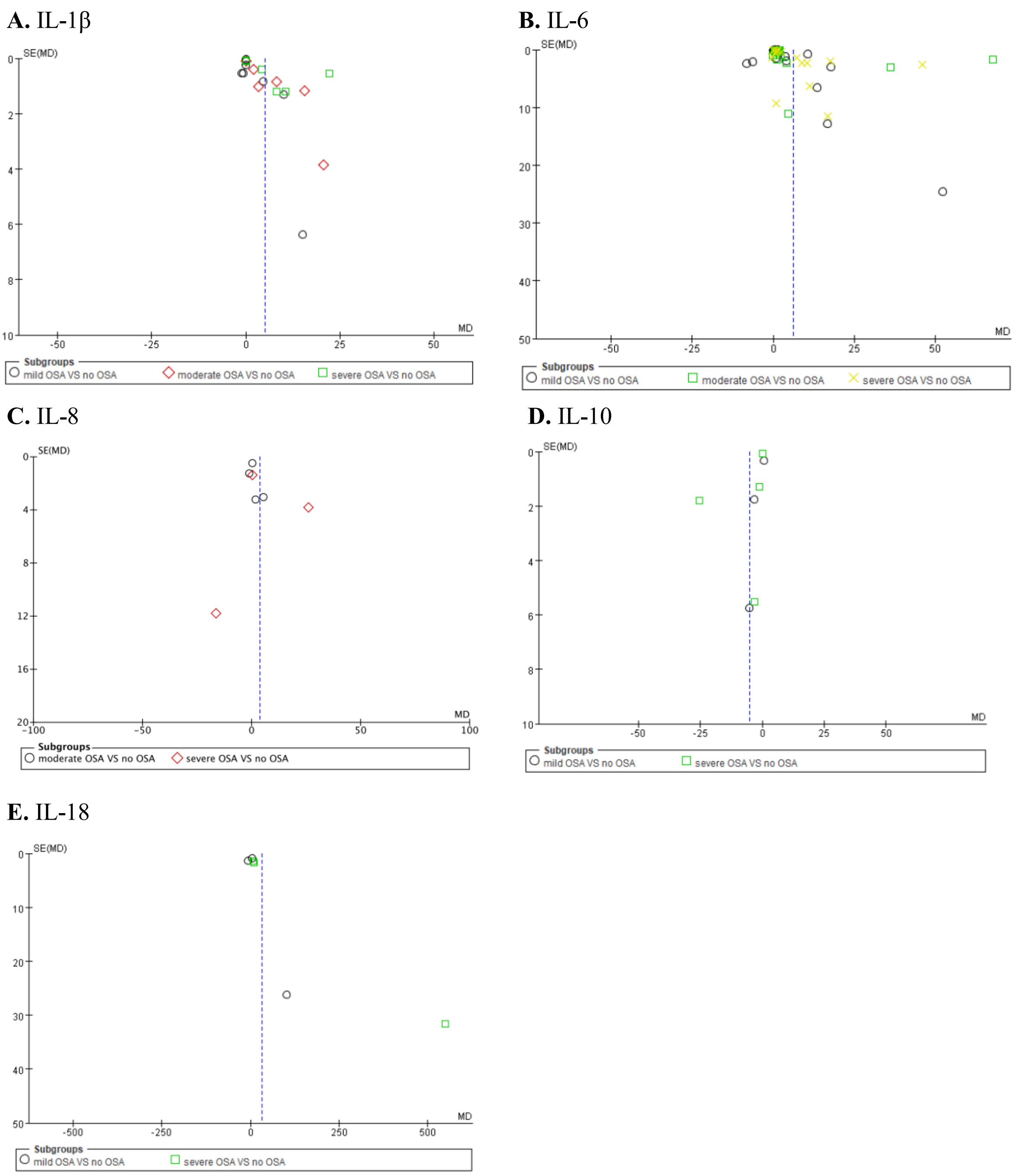

Supplemental Figure 5. Funnel plots of Interleukins (ILs) concentration differences between mild, moderate, severe OSA patients and controls. (A) IL-1β; (B) IL-6; (C) IL-8; (D) IL-10; (E) IL-18.

A. IL-1β

Study or Subgroup	post Mean	post SD	post Total	pre Mean	pre SD	pre Total	Weight	Mean Difference IV, Random, 95% CI
Tirado R. 2017	0.46	0.47	66	0.67	0.61	66	40.3%	-0.21 [-0.40, -0.02]
Chuang H.H. 2020	0.3	0.2	60	0.8	0.8	60	39.2%	-0.50 [-0.71, -0.29]
Huang Y.S. 2020	0.35	0.29	55	1.51	2.25	55	20.5%	-1.16 [-1.76, -0.56]
**Total (95% CI)**			**181**			**181**	**100.0%**	**-0.52 [-0.89, -0.15]**

Heterogeneity: Tau² = 0.08; Chi² = 10.99, df = 2 (P = 0.004); I² = 82%
Test for overall effect: Z = 2.76 (P = 0.006)

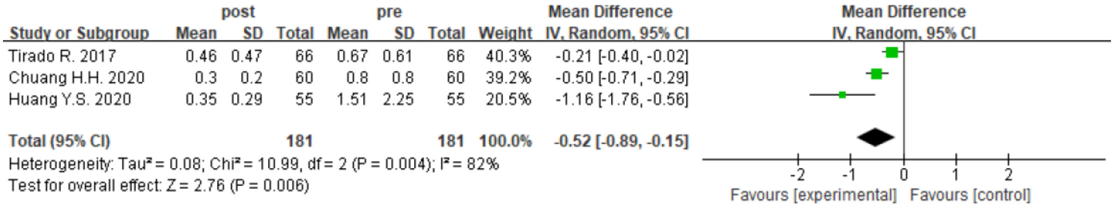

B. IL-6

Study or Subgroup	post Mean	post SD	post Total	pre Mean	pre SD	pre Total	Weight	Mean Difference IV, Random, 95% CI
Arnardottir E.S. 2015	2.8	2	177	2.4	1.7	177	5.5%	0.40 [0.01, 0.79]
Recoquillon S. 2019	1.3	1.2	55	1.1	0.5	55	5.5%	0.20 [−0.14, 0.54]
Dorkova Z. 2008	1.28	1.05	16	1.25	1.1	16	5.5%	0.03 [−0.72, 0.78]
Li A. M. 2008	0.2	0.3	16	0.3	0.4	16	5.5%	−0.10 [−0.34, 0.14]
Campos−Rodriguez F. 2019	3.5	2.6	120	3.6	2.8	120	5.5%	−0.10 [−0.78, 0.58]
Akinnusi M. 2013	1.48	0.97	25	1.71	0.99	25	5.5%	−0.23 [−0.77, 0.31]
Steiropoulos P. 2009	2.46	1.08	32	2.71	1.27	32	5.5%	−0.25 [−0.83, 0.33]
Tirado R. 2017	0.39	0.58	66	0.64	0.69	66	5.6%	−0.25 [−0.47, −0.03]
Huang Y.S. 2020	1.22	1.21	55	1.59	1.58	55	5.5%	−0.37 [−0.90, 0.16]
Salman M.A. 2020	1.8	0.45	79	2.27	0.4	79	5.6%	−0.47 [−0.60, −0.34]
Ryan S. 2006	4.46	16.39	49	5.13	22.65	49	2.0%	−0.67 [−8.50, 7.16]
Kezirian E.J. 2010	2	1.3	21	2.96	2.74	21	5.3%	−0.96 [−2.26, 0.34]
Borges Y.G. 2020	7	4.2	18	8	3.1	18	4.7%	−1.00 [−3.41, 1.41]
Takahashi K.I. 2008	0.634	0.619	27	1.68	2.87	27	5.4%	−1.05 [−2.15, 0.06]
Kheirandish−Gozal L. 2015	7.1	4.7	100	9.2	5.4	100	5.3%	−2.10 [−3.50, −0.70]
Bilal N. 2021	55.17	3.74	10	57.98	5.96	10	3.5%	−2.81 [−7.17, 1.55]
Martínez−Cerón E. 2016	8	4.6	26	11.1	4.7	26	4.7%	−3.10 [−5.63, −0.57]
Eun Y.G. 2010	2.4	3.4	51	7.6	10.4	51	4.4%	−5.20 [−8.20, −2.20]
Li Y 2008	62.2	14.2	2	76.3	1.2	2	0.4%	−14.10 [−33.85, 5.65]
Li Y 2008	59.9	13.2	33	75.7	12.3	33	2.6%	−15.80 [−21.96, −9.64]
Li Y 2008	51.6	23.8	5	71.4	15.4	5	0.3%	−19.80 [−44.65, 5.05]
Wang X 2020	18.68	1.33	54	43.31	2.93	54	5.4%	−24.63 [−25.49, −23.77]
Ye L. 2010	66.04	10.05	10	131.66	20.39	10	0.8%	−65.62 [−79.71, −51.53]
**Total (95% CI)**			**1047**			**1047**	**100.0%**	**−3.21 [−4.58, −1.85]**

Heterogeneity: Tau² = 8.75; Chi² = 3223.89, df = 22 (P < 0.00001); I² = 99%
Test for overall effect: Z = 4.61 (P < 0.00001)

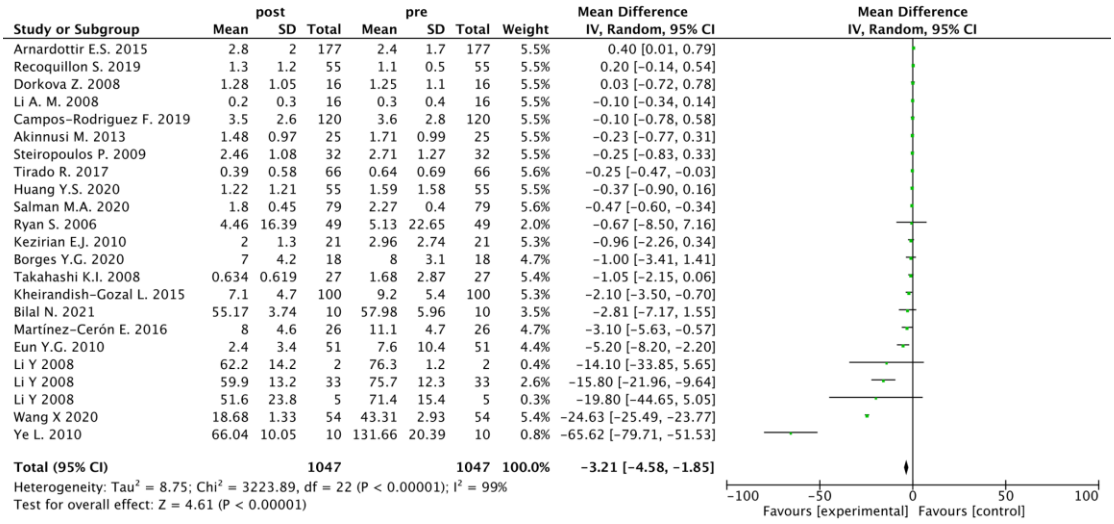

C. IL-8

Study or Subgroup	post Mean	post SD	post Total	pre Mean	pre SD	pre Total	Weight	Mean Difference IV, Random, 95% CI
Akinnusi M. 2013	4.73	1.56	25	4.98	1.67	25	15.6%	-0.25 [-1.15, 0.65]
Li A. M. 2008	1.1	0.6	16	1.9	1	16	16.0%	-0.80 [-1.37, -0.23]
Ryan S. 2006	4.68	1.56	49	5.48	1.53	49	16.0%	-0.80 [-1.41, -0.19]
Martínez-Cerón E. 2016	9.7	4	26	10.7	3.3	26	13.6%	-1.00 [-2.99, 0.99]
Chuang H.H. 2020	4.1	2.2	60	6.4	3.6	60	15.4%	-2.30 [-3.37, -1.23]
Jin F. 2017	35.79	1.63	100	40.72	1.6	100	16.1%	-4.93 [-5.38, -4.48]
Bao H.R. 2005	33.73	8.07	10	38.97	9.72	10	4.1%	-5.24 [-13.07, 2.59]
Bilal N. 2021	112.87	8.99	10	126.31	11.73	10	3.2%	-13.44 [-22.60, -4.28]
**Total (95% CI)**			**296**			**296**	**100.0%**	**-2.22 [-4.06, -0.39]**

Heterogeneity: Tau² = 5.37; Chi² = 209.15, df = 7 (P < 0.00001); I² = 97%
Test for overall effect: Z = 2.38 (P = 0.02)

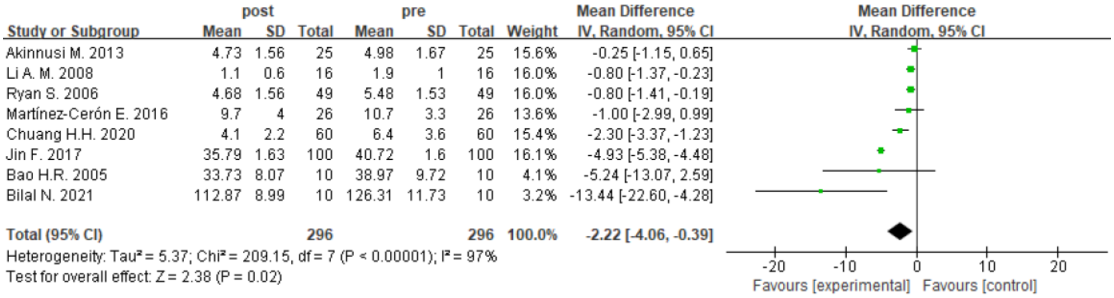

D. IL-10

Study or Subgroup	post Mean	post SD	post Total	pre Mean	pre SD	pre Total	Weight	Mean Difference IV, Random, 95% CI
Huang Y.S. 2020	1.68	1.05	55	2.74	2.94	55	17.9%	-1.06 [-1.89, -0.23]
Chuang H.H. 2020	0.4	0.2	60	1.2	2.1	60	20.4%	-0.80 [-1.33, -0.27]
Ryan S. 2006	1.08	0.6	49	1.31	0.49	49	22.3%	-0.23 [-0.45, -0.01]
Borges Y.G. 2020	4	3.5	18	4	1.6	18	10.1%	0.00 [-1.78, 1.78]
Akinnusi M. 2013	1.03	0.62	25	0.96	0.48	25	21.9%	0.07 [-0.24, 0.38]
Bao H.R. 2005	39.39	3.78	10	33.91	4.29	10	3.8%	5.48 [1.94, 9.02]
Li Y 2008	44.3	5	2	36.1	13.2	2	0.1%	8.20 [-11.36, 27.76]
Li Y 2008	44.9	7.3	33	33.8	8.7	33	3.3%	11.10 [7.23, 14.97]
Li Y 2008	50.9	12.2	5	33.2	8.2	5	0.3%	17.70 [4.82, 30.58]
**Total (95% CI)**			**257**			**257**	**100.0%**	**0.25 [-0.50, 1.01]**

Heterogeneity: Tau² = 0.65; Chi² = 62.77, df = 8 (P < 0.00001); I² = 87%
Test for overall effect: Z = 0.66 (P = 0.51)

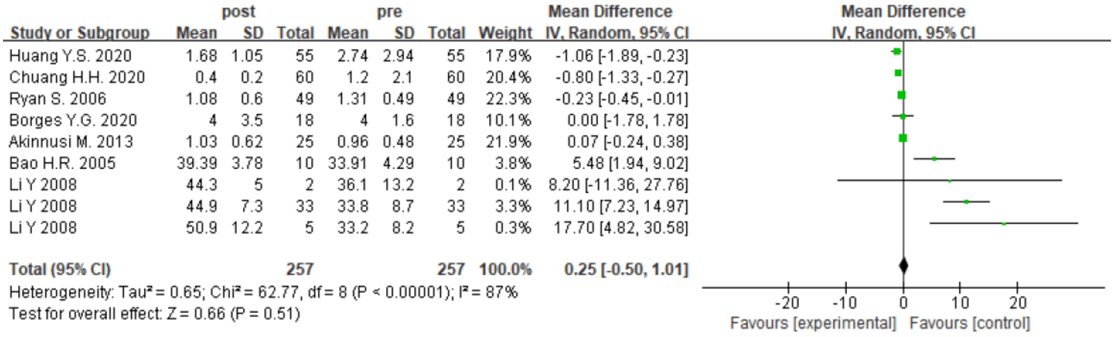

Supplemental Figure 6. Forest plots of interleukins (ILs) treatment concentration differences between post and pre OSA patients. (A) IL-1β; (B) IL-6; (C) IL-8; (D) IL-10

A. IL-1β

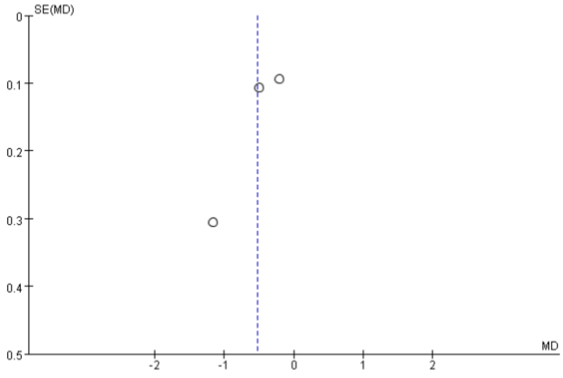

B. IL-6

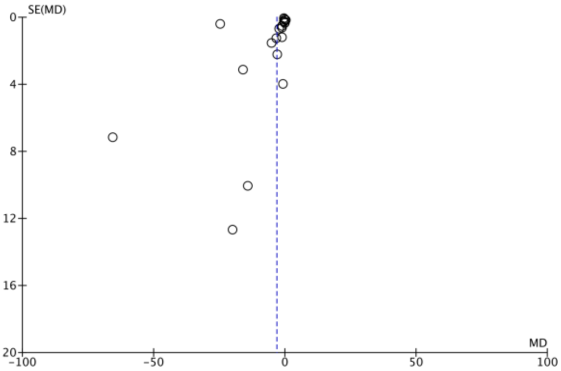

C. IL-8

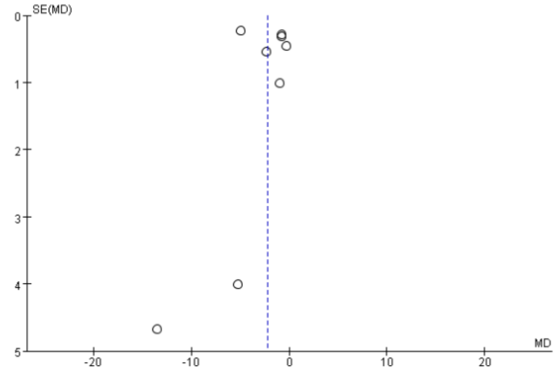

D. IL-10

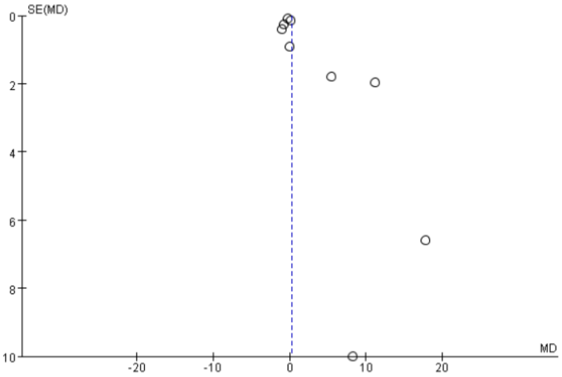

Supplemental Figure 7. Funnel plots of Interleukins (ILs) treatment concentration differences between post and pre OSA patients. (A) IL-1β; (B) IL-6; (C) IL-8; (D) IL-10.

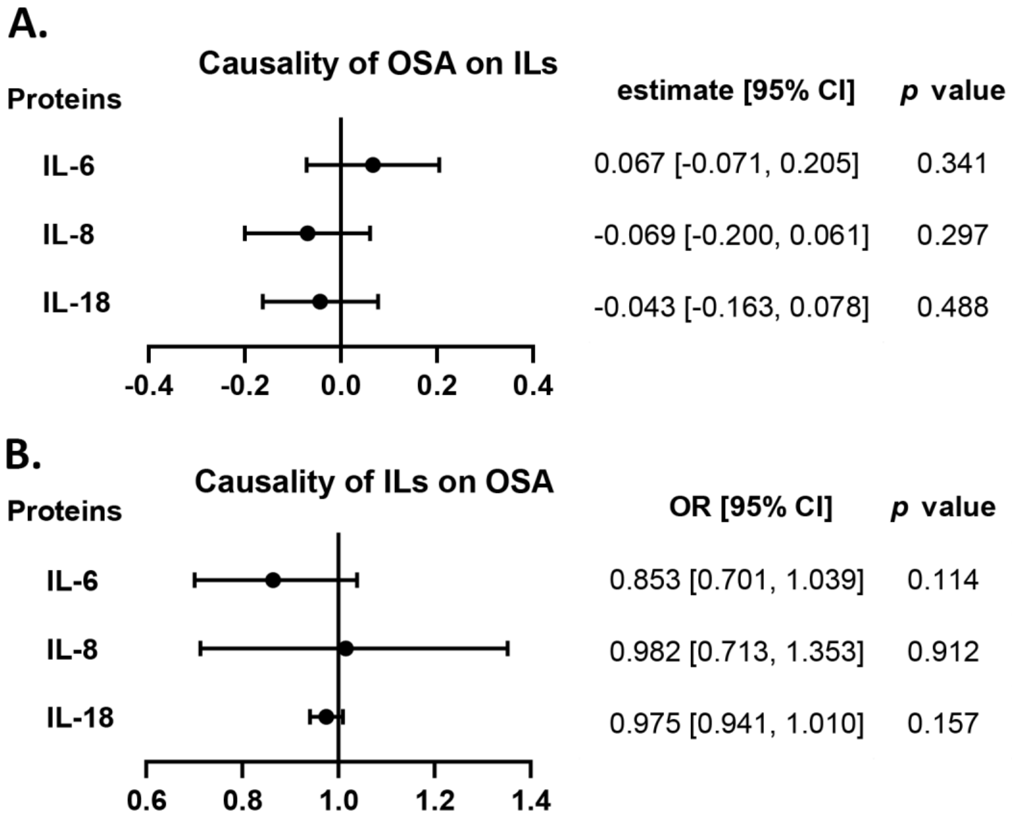

Supplemental Figure 8. Verification of the negative results about the causal relationships between OSA and Interleukins (ILs) of IL-6, IL-8 and Il-18 using another large-sample GWAS

(A). Causality was analyzed with OSA as exposure, IL-6, IL-8, IL-18 individually as outcomes by MR.

(B). Causality was analyzed with IL-6, IL-8, IL-18 respectively as exposure and OSA as outcome by MR. The presented results were performed by method of inverse variance weighted (IVW). The statistically threshold was $p<0.05$.
